# Molecular Phylogeny of Grassland Caterpillars (Lepidoptera: Lymantriinae: *Gynaephora*) Endemic to the Qinghai-Tibetan Plateau

**DOI:** 10.1371/journal.pone.0127257

**Published:** 2015-06-08

**Authors:** Ming-Long Yuan, Qi-Lin Zhang, Zhao-Feng Wang, Zhong-Long Guo, Gen-Sheng Bao

**Affiliations:** State Key Laboratory of Grassland Agro-Ecosystems, College of Pastoral Agricultural Science and Technology, Lanzhou University, Lanzhou, Gansu, People's Republic of China; Institut National de la Recherche Agronomique (INRA), FRANCE

## Abstract

*Gynaephora* (Lepidoptera Erebidae: Lymantriinae) is a small genus, consisting of 15 nominated species, of which eight species are endemic to the Qinghai-Tibetan Plateau (QTP). In this study, we employed both mitochondrial and nuclear loci to infer a molecular phylogeny for the eight QTP *Gynaephora* spp. We used the phylogeny to estimate divergence dates in a molecular dating analysis and to delimit species. This information allowed us to investigate associations between the diversification history of the eight QTP species and geological and climatic events. Phylogenetic analyses indicated that the eight QTP species formed a monophyletic group with strong supports in both Bayesian and maximum likelihood analyses. The low K2P genetic distances between the eight QTP species suggested that diversification occurred relatively quickly and recently. Out of the eight species, five species were highly supported as monophyletic, which were also recovered by species delimitation analyses. Samples of the remaining three species (*G*. *aureata*, *G*. *rouergensis*, and *G*. *minora*) mixed together, suggesting that further studies using extensive population sampling and comprehensive morphological approaches are necessary to clarify their species status. Divergence time estimation results demonstrated that the diversification and speciation of *Gynaephora* on the QTP began during the late Miocene/early Pliocene and was potentially affected by the QTP uplift and associated climate changes during this time.

## Introduction

The Qinghai-Tibetan Plateau (QTP) is the highest (approximately 4500 m above sea level (asl) on average) and one of the most extensive (2.5 × 10^6^ km^2^) plateaus on Earth [1]. The QTP is an economically important region for animal husbandry and is also a biodiversity hotspot [2]. The uplift of the QTP is thought to have begun with the Indian-Eurasian collision about 50 million years ago (Ma) [3–5] and the QTP has gone through several uplift events since the Miocene period (~23 Ma). The emergence of the QTP has largely re-shaped the climate system of central and eastern Asia and produced complex habitats [6–8]. It is widely accepted that the dramatic climatic and environmental shifts caused by the uplift of the QTP are the most important drivers of genetic diversity and divergence patterns in many species in the region [9–12]. Therefore, the QTP has been recognized as a natural laboratory for the study of speciation and biogeography.

Recently, molecular data have been widely used to reconstruct phylogenetic relationships among various taxonomic levels, and to explore the causal correlations between speciation and geological events. Several studies of this nature have been conducted on QTP species, including fishes [13–16], plants [9,10,17–21], amphibians [22–25], birds [26], and mammals [27–29]. These studies suggest that speciation events within these groups occurred during several uplifts of the QTP since Miocene (~23 Ma). Most of this previous work has focused on patterns of diversification in plant and vertebrate species, but invertebrates (e.g., insects) tend to have different life history characteristics (e.g., high population density and short life history) that result in unique patterns of diversification and evolution [30]. The potential effects of the QTP uplift on speciation in QTP insects have never been studied.

The genus *Gynaephora* (Insecta: Lepidoptera: Erebidae: Lymantriinae), also known as grassland caterpillars, was described by Hübner (1822), and the type species is *Gynaephora selenitica* Esper (1783). Currently, this genus contains 15 species, mainly distributed in mountainous areas of the Northern Hemisphere and the Arctic tundra [31,32]. In China, only one species (*G*. *alpherakii*) was known before the 1970s, but seven species were recently described by Chou and Ying (1979) and Liu et al. (1994) based on morphological characteristics [33,34]. The *Gynaephora* species of China are endemic to the QTP (Table 1), and are among the most damaging insect pests to the flora of the QTP alpine meadows [32]. In 2003, the grassland areas damaged by grassland caterpillars were over one million hm^2^ in Qinghai Province of China, leading to the loss of over 90 million CNY [32]. During outbreaks, larvae populations (generally 200–500/m^2^, but the number may exceed 1,000/m^2^) can devour all aboveground herbage, leading to serious shortage of fodder, changing plant community structure, aggravating grassland degeneration and environmental deterioration, and increasing the mortality rate of overwintering livestock and wildlife [32]. More importantly, the cocoons of grassland caterpillars remain in the meadow and can cause skin irritations and blisters, which result in mouth sores and broken tongue disease in domestic animals and wildlife, preventing the animals from foraging and eventually leading to their death [35].

**Table 1:** Original descriptions, type localities, and distributions of the *Gynaephora* species endemic to the Qinghai-Tibetan Plateau.

Species	Original paper	Type locality*	Distribution, habitat & altitude ^#^
*Gynaephora alpherakii* Grum-Grschimailo	[74]	Amdo County, Tibet	Montane meadow; Tibet; ~5000 m asl
*G*. *qinghaiensis* Chou et Ying	[33]	Yushu County, Qinghai Province	Montane meadow; widely distributed in Tibet, Qinghai Province, Sichuan Province, and Gansu Province; from 3000 to 4000 m asl
*G*. *aureata* Chou et Ying	[33]	Zeku County, Qinghai Province	Montane meadow; widely distributed in Qinghai Province and Gansu Province; ~ 3500 m asl
*G*. *menyuanensis* Yan et Chou	[34]	Menyuan County, Qinghai Province	Montane meadow; mainly distributed in the North of Qinghai Province and the Southwest of Gansu Province; no hydrotaxis; from 2900 to 3700 m asl
*G*. *ruoergensis* Chou et Ying	[33]	Ruoergai County, Sichuan Province	Montane meadow; restricted to the Ruoergai Grassland of Sichuan Province; ~ 3500 m asl
*G*. *minora* Chou et Ying	[33]	Ruoergai County, Sichuan Province	Montane meadow; restricted to the Ruoergai Grassland of Sichuan Province; ~ 3500 m asl
*G*. *qumalaiensis* Yan et Chou	[34]	Qumalai County, Qinghai Province	Montane meadow; restricted to Qumalai County, Zhiduo County, and Zaduo County of Qinghai Province; hydrotaxis; from 4000 to 4500 m asl
*G*. *jiuzhiensis* Yan et Chou	[34]	Jiuzhi County, Qinghai Province	Montane meadow; restricted to Jiuzhi County, Dari County, and Gande County of Qinghai Province; hydrotaxis; from 3600 to 4000 m asl

* The type locality of *Gynaephora* are cited from original paper, expect for *G*. *alpherakii* whose type locality is from [74].

^#^ The information is cited from [34] and [31].

Studies on grassland caterpillars are limited, in spite of their impact on the QTP economy and ecology [32]. Several new *Gynaephora* species have been described since 1979, but the phylogeny and evolutionary history of these species have never been estimated. Furthermore, it is crucial to examine whether the current taxonomy of these eight species can be recovered by molecular approaches, as most species have very similar morphological characteristics. These information can be used to gain insight into the speciation process and to make effective pest management strategies.

In this study, we used both mitochondrial and nuclear loci to investigate the molecular phylogeny and diversification history of the eight *Gynaephora* spp. endemic to the QTP. The specific goals of this study were to: (1) assess the taxonomic status of currently recognized *Gynaephora* species on the QTP, (2) investigate the phylogenetic relationships among the eight species of *Gynaephora* in China, and (3) examine associations between the diversification history of the eight QTP species and geological events that formed the QTP.

## Materials and Methods

### Ethics statement

No specific ethics permits were required for the described studies. The insect specimens were collected from alpine meadow of the QTP. No specific permissions were required for these locations/activities. The species in our study are agricultural pests and are not included in the ‘‘List of Protected Animals in China”.

### Sample collection

A total of 145 *Gynaephora* specimens were collected from 15 sampling localities (S1 Table), including all eight described species that occur on the QTP. All specimens were collected in the field and immediately frozen in liquid nitrogen, and stored at -80°C. The habitat features for each *Gynaephora* species endemic to the QTP are shown in Table 1. Two species (*Hyphantria cunea* and *Estigmene acrea*) from Arctiinae and four species (*Lymantria dispar*, *L*. *monacha*, *Orgyia antique* and *O*. *leucostigma*) from Lymantriinae were chosen as outgroup taxa in phylogenetic analyses (S1 Table). Recent molecular phylogenetic analyses have indicated that the subfamilies Lymantriinae and Arctiinae are within Erebidae [36]. We also included three non-QTP *Gynaephora* spp. (*G*. *selenitica*, *G*. *rossii*, and *G*. *groenlandica*) to infer the phylogenetic relationships within the genus *Gynaephora*. DNA sequences of outgroup taxa were attained from GenBank (S1 Table). There is no molecular data available from GenBank for the remaining four *Gynaephora* spp. These species have not been reported recently and are endemic to Europe, Russia and Hindu Kush Mountain (*G*. *lugens*), Altai Plateau of Russia (*G*. *pumila*), Pamirs, Kunlun Montain and Ukraine (*G*. *selenophora*), and West of Pamirs (*G*. *sincera*) [31].

### DNA extraction, PCR and sequencing

Total genomic DNA was extracted from individual specimens using the Genomic DNA Extraction Kit (TIANGEN, Beijing, China) according to the manufacturer’s protocol. We amplified and sequenced partial sequences of two mitochondrial genes (mitochondrial cytochrome oxidase subunit 1 [COI] and NADH dehydrogenase subunit 5 [ND5]) and two nuclear genes (glyceraldehyde-3-phosphate dehydrogenase [GAPDH] and elongation factor 1-alpha [EF-1α]) for all 145 individuals (S1 Table). Universal insect primers for COI and two nuclear genes were obtained from [37] and [38], respectively, and *Gynaephora*-specific ND5 primers were designed in this study (Table 2). Each PCR reaction was performed in a total volume of 25 μL, containing 2.5 μL of 10x PCR reaction buffer (with Mg^2+^), 1.5 μL of dNTPs (each 2.5 mM), 1.5 μL of each of two primers (20 μM), 2 μL of the extracted DNA, 0.2 μL Taq DNA polymerase (5 U/μL, TAKARA), and 16.8 μL of distilled water. The PCR conditions were as follows: 94°C for 5 min, 35 cycles of 94°C for 30 s, a primer-specific annealing temperature of 46–50°C (Table 2) for 50 s, 72°C for 50 s, and a final extension at 72°C for 10 min. All PCR products were separated by electrophoresis on a 1.2% agarose gel, purified with a DNA gel purification kit (Omega, USA), and sequenced in both directions on an ABI3730 automated sequencer (Sangon Biotech, Shanghai, China). The PCR primers were also used for sequencing.

**Table 2:** PCR primers used in the present study.

Gene name	Primer name	Primer sequence (5'-3')	Annealing temperature (°C)	Reference
COI	LCO1490	GGTCAACAAATCATAAAGATATTGG	46	[37]
	HC02198	TAAACTTCAGGGTGACCAAAAAATCA		[37]
ND5	ND5-F	CCCCCTATATAACGAATATCTTG	46	This study
	ND5-R	TTAGGTTGGGATGGTTTAGG		This study
GAPDH	GAPDH-F	TAATACGACTCACTATAGGGAARGCTGGRGCTGAATATGT	48	[38]
	GAPDH-R	ATTAACCCTCACTAAAGGWTTGAATGTACTTGATRAGRTC		[38]
EF-1α	EF1-F	TAATACGACTCACTATAGGGCACATYAACATTGTCGTSATYGG	50	[38]
	EF1-R	ATTAACCCTCACTAAAGCATRTTGTCKCCGTGCCARCC		[38]
	EF2-F	TAATACGACTCACTATAGGGGAGGAAATYAARAARGAAG	50	[38]
	EF2-R	ATTAACCCTCACTAAAGACAGCVACKGTYTGYCTCATRTC		[38]

### Sequence data exploration

DNA sequences were aligned for each gene, independently using ClustalW (codons) implemented in MEGA 5.10 [39] with the default parameters. Sequences were examined for the presence of stop codons or indels, which could reveal pseudogene sequences. Identical haplotypes were collapsed using DNASP 5.10 [40]. For each gene, transitions and transversions were plotted against sequence divergence in DAMBE 5.0.59 [41] to evaluate the possibility of sequence saturation. Since there was no evidence of saturation in the gene sequences, all codon positions were included in the analyses. The numbers of haplotypes and standard diversity indices [haplotype (*h*) and nucleotide (*π*) diversities] for each gene were estimated using DNASP 5.10 [40]. The pairwise genetic distances between species were calculated in MEGA 5.10 [39] with a Kimura 2-parameter (K2P) model.

### Phylogenetic analyses

Maximum likelihood (ML) and Bayesian inference (BI) phylogenetic trees were estimated for each individual mitochondrial gene, the combined mitochondrial gene dataset (COI and ND5), the combined nuclear gene dataset (EF-1α and GAPDH), as well as a concatenated dataset of all of the genes. The PartitionFinder 1.1.1 [42] was used to find the best partitioning schemes and corresponding nucleotide substitution models for each dataset. We defined data blocks based on genes and/or codon positions, and used the Bayesian information criterion (BIC) and the ‘‘greedy” algorithm with branch lengths estimated as ‘‘unlinked” to search for the best-fit scheme (S2 Table). The best-fit partitioning schemes and models determined by PartitionFinder were used for the subsequent analyses. Both BI and ML tree constructions were performed on the CIPRES Science Gateway 3.3 [43]. ML analyses were conducted with RAxML-HPC2 on XSEDE 8.0.24 [44] using GTRGAMMA model, and 1,000 bootstraps (BS) were used to estimate the node reliability. BI analyses were performed with MrBayes 3.2.3 [45] on XSEDE. For each dataset, two independent analyses starting from different a random tree were run in parallel for ten million generations, sampling every 1,000 generations. Stationarity is considered to be reached when ESS (estimated sample size) value is above 100 and PSRF (potential scale reduction factor) approach 1.0 as MrBayes 3.2.3 suggested [45]. The first 25% of samples were discarded as burn-in, and the remaining trees were used to calculate posterior probabilities (PP) in a 50% majority-rule consensus tree.

### Species delimitation

We conducted species delimitation analyses using three datasets (the COI dataset, the ND5 dataset, and the mitochondrial gene dataset) with the following two approaches. First, we used Automatic Barcode Gap Discovery (ABGD) [46] to discover candidate species. This method is an automatic procedure to partition the dataset into putative species based on the barcode gap without an a priori species hypothesis [46]. We submitted three datasets to the ABGD online website (http://wwwabi.snv.jussieu.fr/public/abgd/abgdweb.html), and run with the default parameters. Both Jukes-Cantor (JC69) and Kimura (K80) models were tested.

A second species delimitation analysis was performed with the Poisson Tree Processes (PTP) including bayesian implementation of the model [47]. Analyses were conducted on the bPTP web Server (http://species.h-its.org/ptp/) using rooted phylogenetic trees from RAxML analyses. We used the following parameters: MCMC, 500,000 generations; Thinning, 100; Burnin, 0.1; Seed, 123; and removed outgroups.

### Molecular dating

Divergence times in the *Gynaephora* phylogeny were estimated using the four gene dataset in BEAST 1.8.0 [48] on the CIPRES Science Gateway 3.3 [43]. As there is no reliable lymantriine fossil record that could be used to calibrate the tree, the secondary calibration approach was used with caution here. To use the temporal framework of Toussaint *et al*. [49] and Wahlberg *et al*. [50], additional 33 taxa from the families Erebidae and Nolidae were included in our dataset (S1 Table). For Nolidae, six taxa were taken from Toussaint *et al*. [49] and Wahlberg *et al*. [50]. For Erebidae, additional 27 taxa representing seven subfamilies were selected according to the results of recent molecular phylogenetic analyses of Erebidae [36]. Two temporal constraints were imposed on the tree to estimate the divergence time. Both were obtained from Wahlberg *et al*. [50], which utilized six fossil calibrations and one secondary calibration point with extensive taxa sampling and accounted for uncertainty around each point. The crown ages of both Erebidae and Nolidae were set as a normal distribution with a mean of 55 Ma and a standard deviation of 5 Ma.

The BEAST.xml files were created in BEAUti 1.8.1 [48] with the following settings: the “Site Model” was set as GTR+I+G model proposed by the jModeltest 2.1.3 [51], the “Clock Model” was set to a strict clock or a relaxed-clock with uncorrelated rates, the “Tree Models” were set to a Yule or Birth-Death process of speciation, the MCMC chain length was set to 3 × 10^9^ generations with a 10% burn-in, and the remaining parameters used default settings. BEAST analyses were repeated three times. The best fit models for “Clock model” and “Tree model” were selected with a Bayes Factor (BF) approach, and the log marginal likelihood values were calculated using path sampling (PS, [52]) and stepping-stone sampling (SS, [53]) [54,55]. Chain convergence was assessed by examining the effective sample size (ESS) of parameters with Tracer 1.5 (http://tree.bio.ed.ac.uk/software/tracer/). The 95% highest posterior densities (95% HPD) and 50% majority rule consensus trees were summarized using TreeAnnotator 1.8.1 [48]. Trees were visualized using the FigTree 1.4.2 (http://tree.bio.ed.ac.uk/software/figtree/).

## Results

### Sequence data

We obtained 3,196 bp sequences for each individual, including 1,271 bp mitochondrial (COI, 658 bp; ND5, 613 bp) and 1,925 bp nuclear (GAPDH, 685 bp; EF-1α, 1,240 bp) sequences (Table 3, S1 Table). A total of 580 sequences were deposited in GenBank under accession numbers KF887501–KF887904 and KP419744–KP419919 (S1 Table). No length polymorphisms or stop codons were observed in the four protein-coding genes in any of the studied specimens. Sequence polymorphism data for each gene were presented in Table 3. Compared to the two mitochondrial genes, two nuclear genes showed markedly low genetic polymorphisms, with only 16 variable sites and 13 parsimony informative sites.

**Table 3:** Sequence polymorphism data.

Dataset	*N*	Size (bp)	VS (%)	PIS (%)	*n*	*h* ± SD	*π* ± SD (%)
COI	145	658	47 (7.14)	46 (6.99)	12	0.895 ± 0.007	2.457 ± 0.054
ND5	145	613	34 (5.55)	34 (5.55)	12	0.899 ± 0.007	1.931 ± 0.045
GAPDH	145	685	8 (1.17)	7 (1.02)	10	0.707 ± 0.032	0.155 ± 0.011
EF-1α	145	1,240	8 (0.65)	6 (0.48)	10	0.681 ± 0.026	0.083 ± 0.007
Mitochondrial dataset (COI + ND5)	145	1,271	81 (6.37)	80 (6.29)	21	0.922 ± 0.008	2.203 ± 0.047
Nuclear dataset (GAPDH + EF-1α)	145	1,925	16 (1.66)	13 (0.78)	26	0.877 ± 0.017	0.109 ± 0.006
Combined gene dataset (COI + ND5 + GAPDH + EF-1α)	145	3,196	97 (3.54)	93 (2.97)	58	0.974 ± 0.004	0.942 ± 0.020

*N*, number of individuals sequenced; VS, variable sites; PIS, parsimony information sites; *n*, number of different haplotype; *h*, haplotype diversity; and *π*, nucleotide diversity.

A total of 12, 12, 10, and 10 haplotypes were identified in COI, ND5, GAPDH, and EF-1α sequences, respectively (Table 3, S1 Table). Some of the haplotypes were shared between different species, and this was especially evident in the two nuclear genes. When the four gene sequences were combined, a total of 58 unique haplotypes were identified. These haplotypes were unique to species, except for three haplotypes which were shared between *G*. *rouergensis* and *G*. *minora* (S1 Table). The K2P genetic distances between pairs of species were listed in Table 4. The K2P distance values of COI between *G*. *selenitica* and other *Gynaephora* spp. were the highest (8.96–11.03%), and the lowest value (0.29%) was found between two QTP species (*G*. *ruoergensis* and *G*. *minora*). For the combined four gene dataset, the K2P distances among the eight QTP *Gynaephora* spp. were markedly low (0.10–1.78%).

**Table 4:** Means of Kimura 2-parameter genetic distances (%) between *Gynaephora*.

Species	1	2	3	4	5	6	7	8	9	10	11
1 *G*. *alpherakii*	**0**	1.28	1.48	1.27	1.22	1.42	1.35	1.21	–	–	–
2 *G*. *aureata*	3.46	**0.35**	1.49	0.50	0.15	1.67	1.42	0.15	–	–	–
3 *G*. *jiuzhiensis*	4.04	4.74	**0.08**	1.58	1.47	0.40	0.42	1.46	–	–	–
4 *G*. *menyuanensis*	3.46	1.38	4.73	**0**	0.49	1.78	1.60	0.48	–	–	–
5 *G*. *minora*	3.42	0.38	4.69	1.34	**0.34**	1.66	1.41	0.10	–	–	–
6 *G*. *qinghaiensis*	3.63	4.67	0.72	4.66	4.62	**0**	0.44	1.65	–	–	–
7 *G*. *qumalaiensis*	3.65	4.00	0.74	4.34	3.96	0.67	**0.04**	1.40	–	–	–
8 *G*. *ruoergensis*	3.32	0.33	4.59	1.19	0.29	4.52	3.85	**0.17**	–	–	–
9 *G*. *groenlandica*	8.38	8.80	8.13	8.42	8.75	8.06	7.67	8.64	**0.33**	–	–
10 *G*. *rossii*	8.95	8.81	8.45	8.40	8.77	8.38	7.99	8.66	5.81	**0.62**	–
11 *G*. *selenitica*	11.03	10.63	10.31	10.07	10.58	10.24	9.52	10.46	9.58	8.96	**0.16**

Data above the diagonal are interspecific genetic distances based on the combined four genes (COI, ND5, GAPDH and EF-1α), with COI distances below. Data in bold (the diagonal) are intraspecific COI distances.

### Phylogenetic relationships

**Fig 1:**
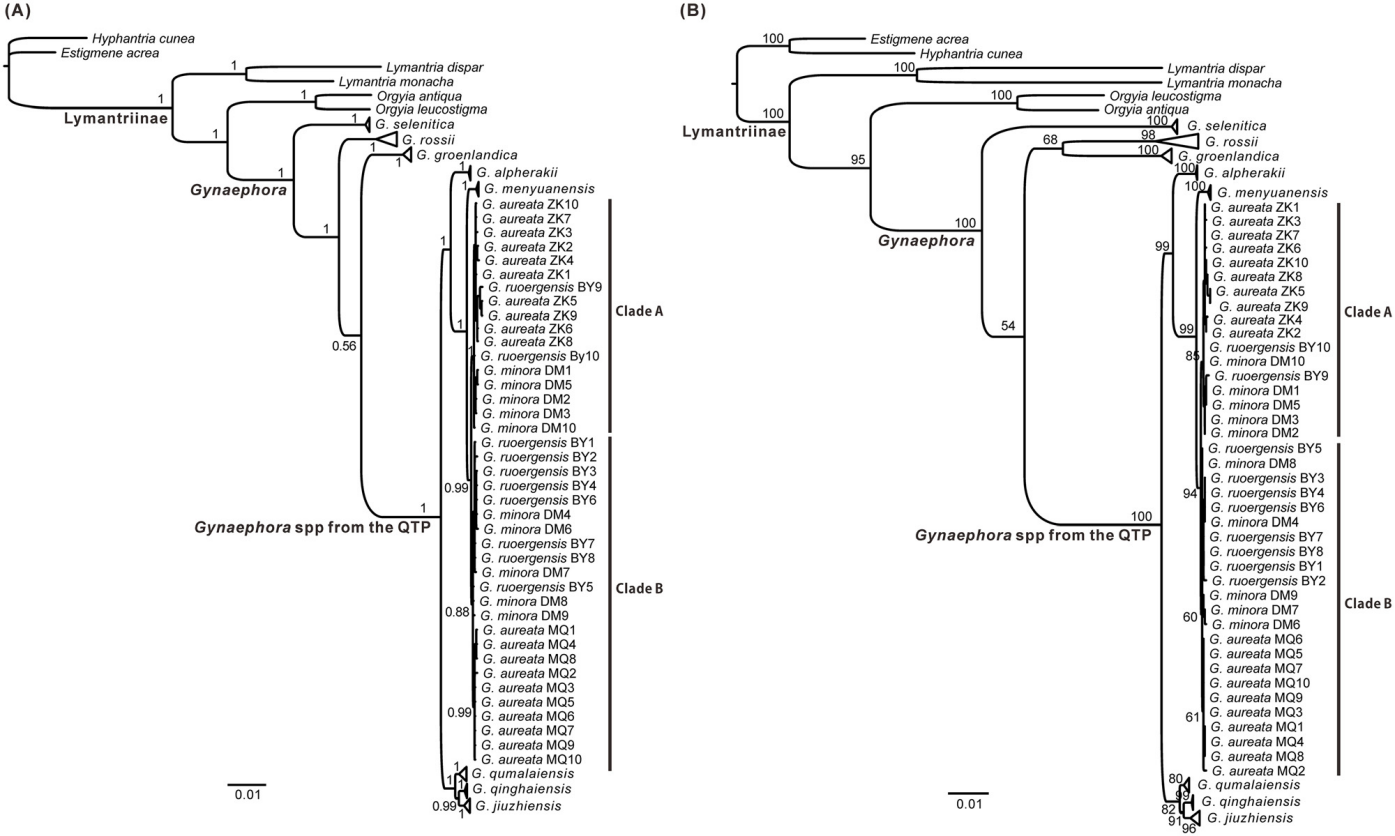
Phylogenetic trees of *Gynaephora* based on the concatenated sequences of two mitochondrial (COI and ND5) and two nuclear (GAPDH and EF-1α) genes. (A) Bayesian tree. Numbers at nodes indicate Bayesian posterior probabilities (PP). (B) Maximum tree. Numbers at nodes indicate bootstrap support values (BS). For full phylogenetic trees see S1 and S2 Figs.

Phylogenetic analyses with five datasets and two inference methods resulted in almost identical tree topologies, with slight differences occurring in some nodes poorly supported and BI trees having higher supports for internal branches (Fig 1, S1 and S2 Figs). The eight *Gynaephora* spp. from the QTP formed a strongly supported monophyletic group in all BI and ML trees (PP = 0.99–1.0, BS = 100). The analyses based on the combined nuclear dataset generated poorly resolved trees, and none of the eight *Gynaephora* spp. were supported as monophyletic, potentially caused by the limited number of parsimony informative sites (Table 3). Phylogenetic analyses of ND5 produced relatively good resolution, but only four of the *Gynaephora* spp. were recovered as monophyletic group in both BI and ML analyses (PP = 1.0, BS = 85–100; S1 and S2 Figs), as found in the BI tree of the COI dataset (PP = 1.0, S1 Fig). In the ML tree of the COI dataset, five monophyletic groups were found, but *G*. *jiuzhiensis* and *G*. *qumalaiensis* were recovered as sister-species with low support (BS = 42, S2 Fig). Analyses of both the mitochondrial gene dataset and the four gene combined dataset yielded well-resolved clades with good support (Figs 1a and 1b, S1 and S2 Figs). The eight recognised *Gynaephora* species on the QTP were resolved as two main clades (Fig 1, S1 and S2 Figs). One clade was composed of five species: *G*. *alpherakii*, *G*. *menyuanensis*, *G*. *aureata*, *G*. *rouergensis*, and *G*. *minora*, and the other clade contained three species: *G*. *qinghaiensis*, *G*. *qumalaiensis*, and *G*. *jiuzhiensis*. Five monophyletic clades with strong supports (PP ≥ 0.99, BS ≥ 80) corresponded to five currently recognized species. However, three *Gynaephora* spp. (*G*. *aureata*, *G*. *rouergensis* and *G*. *minora*) were paraphyletic and divided into two clades (Clades A and B; Fig 1). These three species are closely related to *G*. *menyuanensis* (PP = 1.0, BS = 99; Fig 1, S1 and S2 Figs). *G*. *alpherakii* is sister to the other species in the same clade (PP = 1.0, BS = 99; Fig 1, S1 and S2 Figs). *G*. *qinghaiensis* and *G*. *jiuzhiensis* are more closely related to each other (PP = 0.99, BS = 91; Fig 1, S1 and S2 Figs) than to *G*. *qumalaiensis*.

### Species delimitation

The ABGD analyses for the three datasets yielded variable group numbers, depending on different prior threshold, JC69 or K80 distance model, and initial or recursive partitions (Table 5). When two mitochondrial genes were combined together, the ABGD analyses resulted in a stable group count (6) with a range of prior intraspecific values (*P* = 0.0010–0.0077) in both initial and recursive partitions. Among the six groups, five corresponded to the morphologically recognised *Gynaephora* species (S3 Table). The remaining one group consisted of three species (*G*. *aureata*, *G*. *ruoergensis*, and *G*. *minora*), and could be further divided into two groups (i.e., Clades A and B in the phylogenetic analyses) by some ABGD analyses only for the COI dataset (S3 Table).

**Table 5:** Results of ABGD analyses with JC69 distance model.

Prior intraspecific distance (*P*)	No. groups of COI dataset		No. groups of ND5 dataset		No. groups of the mitochondrial gene dataset	
	Initial partition	Recursive partition	Initial partition	Recursive partition	Initial partition	Recursive partition
0.0010	6 (7)	12 (12)	6 (6)	12 (12)	6 (6)	6 (6)
0.0017	6 (7)	7 (7)	6 (6)	6 (6)	6 (6)	6 (6)
0.0028	6 (7)	7 (7)	6 (6)	6 (6)	6 (6)	6 (6)
0.0046	3 (3)	6 (6)	3 (3)	5 (5)	6 (6)	6 (6)
0.0077	3 (3)	3 (3)	3 (3)	3 (3)	6 (6)	6 (6)
0.0129	0	1 (1)	0	1 (1)	0	1 (1)

Values in the bracket are the results obtained with K80 distance model.

Results of bPTP analyses for each the three datasets (COI, ND5 and COI+ND5) were shown in S3 Fig. The bPTP analysis using the COI dataset identified four putative species (PP = 0.51–0.99; S3A Fig), of which two corresponded to the morphologically recognised species, i.e. *G*. *alpherakii* (PP = 0.99) and *G*. *menyuanensis* (PP = 0.76). For the ND5 dataset, eight putative species were recovered (S3B Fig), but only three species were congruent with the morphologically recognised species, i.e. *G*. *qinghaiensis* (PP = 0.92), *G*. *alpherakii* (PP = 0.82) and *G*. *menyuanensis* (PP = 0.54). When two mitochondrial genes were combined together, the bPTP analysis recovered six putative species, of which five were recognised by morphologically characteristics (PP = 0.65–1.0; S3C Fig). Three species (*G*. *qinghaiensis*, *G*. *jiuzhiensis* and *G*. *qumalaiensis*) consistently recovered as a single bPTP group by the three datasets (S3A–S3C Figs).

### Divergence times

**Fig 2:**
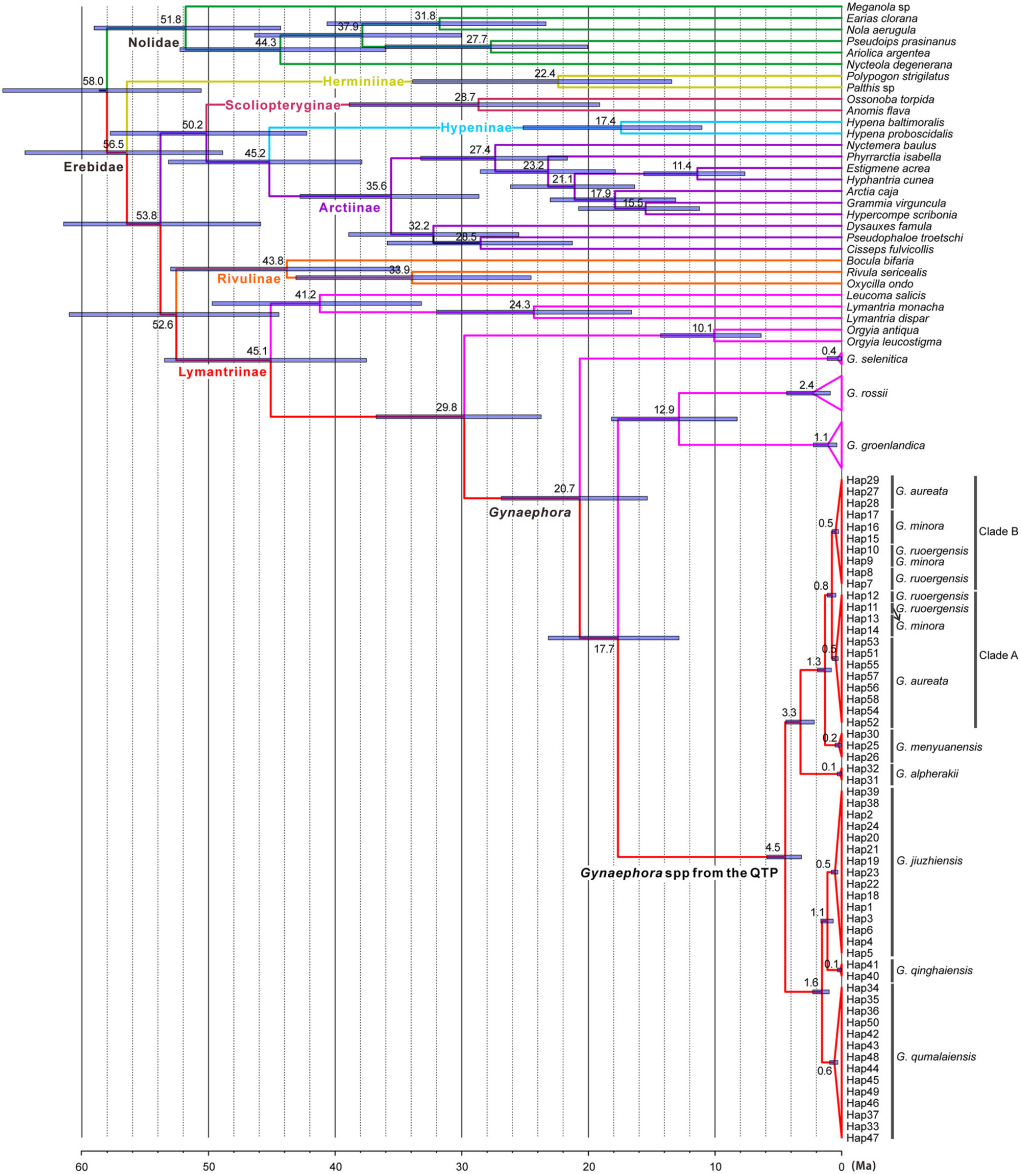
Estimates of divergence times obtained with BEAST. The numbers above nodes are the mean divergence times. The node bars indicated the 95% highest posterior densities of the divergence time estimates. For haplotype information see S1 Table.

All BEAST analyses showed high convergence, with ESS values well above 3,000 for all parameters. Bayes factors indicated that the lognormal relaxed clock was favored compared to the strict clock, and the best fit tree model for our data was the Birth-Death process of speciation (S4 Table). The results for all age estimates with 95% HPD intervals were presented in Fig 2.

The most recent common ancestor of the eleven *Gynaephora* spp. was estimated at approximately 20.7 Ma (95% HPD: 15.4–26.9 Ma). The split between two main clades of the eight QTP *Gynaephora* spp. occurred at about 4.5 Ma (95% HPD: 3.2–5.9 Ma). The early splits within each main clade occurred at 3.3 Ma (95% HPD: 2.2–4.4 Ma) and 1.6 Ma (95% HPD: 1.0–2.3 Ma), respectively. The divergence time between *G*. *jiuzhiensis* and *G*. *qinghaiensis* was 1.1 Ma (95% HPD: 0.7–1.7 Ma). *G*. *menyuanensis* diverged from Clades A and B (*G*. *aureata*, *G*. *rouergensis*, and *G*. *minora*) at 1.3 Ma (95% HPD: 0.8–1.9 Ma), followed by a divergence between Clades A and B at 0.8 Ma (95% HPD: 0.5–1.1 Ma). The intraspecific divergence times were all within 0.6 Ma.

## Discussion

### Phylogenetic relationship and species status of *Gynaephora*

In this study, we employed multi-locus DNA data to estimate the first molecular phylogenetic relationships of *Gynaephora* species, including 11 of 15 species, i.e. all the eight taxa endemic to the QTP and three from other regions including the type species (i.e., *G*. *selenitica*). Phylogenetic analyses based on COI sequences indicated that all the eleven *Gynaephora* spp. included in the current study formed a monophyletic group with high supports (PP = 1.0, BS = 99; S1 and S2 Figs). However, the eight QTP *Gynaephora* spp. is sister to the clade formed by *G*. *rossii* and *G*. *groenlandica* in the Bayesian analysis (PP = 1.0), instead to sister to *G*. *groenlandica* in the ML analysis (BS = 60). The topology incongruence between BI and ML analyses was also observed for other datasets. As no sequences are presently available for the other four *Gynaephora* spp., we do not know yet the complete phylogenetic relationship among the genus *Gynaephora*. However, the eight QTP species consistently formed a monophyletic clade with high supports in phylogenetic analyses (PP = 1.0, BS = 100), regardless of the analytical datasets and methods (Fig 1, S1 and S2 Figs). Additionally, the COI genetic distances between the three non-QTP species and the eight QTP species (8.96–11.03%) much larger than that of within the latter (0.29–4.74%) (Table 4). Therefore, out results preliminarily supported the monophyly of the eight QTP *Gynaephora* spp., though further research including all the 15 *Gynaephora* spp. is needed.

Five species (*G*. *menyuanensis*, *G*. *qinghaiensis*, *G*. *alpherakii*, *G*. *qumalaiensis*, and *G*. *jiuzhiensis*) were recovered as monophyletic in both BI and ML analyses (Fig 1, S1 and S2 Figs), and were also strongly supported as valid species by both ABGD and bPTP analyses (Table 5, S3 Table, S3 Fig) in spite of low K2P genetic distances among the five monophyletic species (K2P_COI_ = 0.67–4.74%, K2P_four genes_ = 0.40–1.78%; Table 4). The gene tree topology based on the mitochondrial gene dataset was most similar to the topology of the combined dataset, with five species recovered as monophyletic (PP ≥ 0.99, PS ≥ 85; Fig 1, S1 and S2 Figs), indicating that mitochondrial genes are useful genetic markers for species delimitation in *Gynaephora*. In contrast, the two nuclear gene trees lacked resolution at the species level and this might be attributed to insufficient phylogenetic information in the EF-1α and GAPDH sequences at this taxonomic level. Our results confirmed that the nuclear DNA markers like these two have slower rate of evolution that is already shown in previous studies [56,57] and are more suitable for deep phylogenetic studies [36,49,58,59].

At least one sample from the type locality was collected for each *Gynaephora* species (Table 1, S1 Table), but *G*. *aureata*, *G*. *rouergensis*, and *G*. *minora* were not supported as monophyletic species. These three species mixed together and were divided into Clades A and B in the phylogenetic trees of the combined dataset, and consistently supported as two potentially distinct species by species delimitation analyses (ABGD and bPTP) based on the mitochondrial gene dataset (Table 5, S3 Table, S3 Fig). These three *Gynaephora* species were recently described by Chou and Ying (1979) and they were recognized as valid species by Yan (2006). They can be differentiated by overall size differences and subtle features of the wing colour and shape of the external genitalia [33]. For example, *G*. *rouergensis* and *G*. *minora* have body lengths of 7 mm and 5 mm, and their wingspan is 27 mm and 12 mm. It is noteworthy that *G*. *rouergensis* is sympatrically distributed with *G*. *minora*, and the two species are restricted to Ruoergai County of Sichuan Province [33]. *G*. *aureata* has a parapatric distribution with *G*. *rouergensis* and *G*. *minora*. Furthermore, some of the haplotypes for each gene were shared among these three species (S1 Table). Therefore, it is plausible that interspecific hybridization might occur between these three species, which could result in extremely low interspecific genetic distances (K2P_COI_ = 0.29–0.38%, K2P_four genes_ = 0.10–0.15%; Table 4), but relatively high intraspecific distances (K2P_COI_ = 0.17–0.35%; Table 4). Considering their similar morphological characteristics and extremely low genetic distances, *G*. *aureata*, *G*. *rouergensis*, and *G*. *minora* might be better described as a species complex, i.e. the *G*. *aureata* complex. This complex was highly supported by phylogenetic analyses (PP = 1.0, BS = 99; Fig 1) and species delimitation analyses (Table 5, S3 Table, S3 Fig). However, our molecular data represent only a small sampling of the genetic data, and further studies using comprehensive morphological characteristics and molecular data (multiple unlinked genes or genomic data) from more populations and individuals are needed to clarify the taxonomic status of these three putative species.

### Divergence patterns of *Gynaephora* on the QTP

Given that all the eight *Gynaephora* spp. from the QTP well formed a monophyletic clade in all phylogenetic analyses (Fig 1, S1 and S2 Figs), *Gynaephora* spp. on the QTP most likely arose from a single origin. Among 15 reported *Gynaephora* spp., more than half of the species are endemic to the QTP [31,32]. An outstanding feature of *Gynaephora* spp. on the QTP is that most species are highly restricted in their distribution (Table 1). Therefore, geographic isolation may play an important role in the speciation process of *Gynaephora* on the QTP. Isolation and subsequent divergence have been proposed as an important mechanism of speciation, as demonstrated by previous studies on some endemic species/genera on the QTP [9,11,12]. Therefore, it is likely that the common ancestor of QTP *Gynaephora* may have been widely distributed in the QTP, and subsequently diverged due to the formation of mountains and valleys accompanying the QTP uplift. This hypothesis best fits the divergence pattern seen in one main clade including three *Gynaephora* spp. (*G*. *jiuzhiensis*, *G*. *qinghaiensis*, and *G*. *qumalaiensis*) which occupy adjacent distribution ranges (Table 1) and are strongly supported as sister species (Fig 1, S1 and S2 Figs). This hypothesis does not adequately explain all of the relationships though. Although *G*. *alpherakii* is geographically close to *G*. *qinghaiensis*, they belong to different main clades (Fig 1). *G*. *alpherakii* is restricted to high altitude environments (~ 4500 m asl) and is geographically separated from the other four species in the same clade that are found in relatively low altitude habitats (~3500 m asl) (Table 1, S1 Table). The biogeographic pattern of QTP *Gynaephora* spp. might be more complicated than we thought, and further analysis with extensive sampling is required to uncover the vicariance, migration, and speciation history of *Gynaephora* spp. on the QTP.

### Association between *Gynaephora* evolution and the uplift of the QTP

Estimating evolutionary timeframe from genetic data is a complex process [60], and calibration has a significant, sometimes drastic impact on estimated divergence time [61]. Although rate of substitution for COI has been widely used in insect divergence time estimates, substitution rates greatly differ among insect lineages [62–64]. Due to the lack of fossil record presently available for the subfamily Lymantriinae, the secondary calibration approach was used with caution in the present study. Our molecular dating results showed that the two calibration nodes were highly supported (PP = 1.0), and the median ages for the two nodes were 51.8 Ma and 56.5 Ma, which were highly congruent with the results of Toussaint *et al*. [49] and Wahlberg *et al*. [50]. Although more taxa of the subfamily Arctiinae were included in our study, the divergence time between *Dysauxes famula* and *Pseudophaloe troetschi* was estimated to be 35.6 Ma, which was highly similar to that of Wahlberg *et al*. [50]. Including the root calibration did not have a large effect on the resulting age estimates. Hence, our BEAST analyses should gave reasonable age estimates for the diversification of the eight QTP *Gynaephora* spp.

Our molecular dating analyses suggested that the eight QTP *Gynaephora* spp. diverged from *G*. *rossii and G*. *groenlandica* at about 17.7 Ma. This time frame corresponds with the conclusion of the initial uplift of the QTP during the Early Miocene (25–17 Ma) [65]. The rapid diversification event that resulted in the split between two main clades occurred around 4.5 Ma and may be associated with accelerated uplift of the QTP during the late Miocene/early Pliocene [4,66]. Further diversification in each main clade gave rise to many extant species and is estimated to have occurred 3.3–1.1 Ma, during an extensive period of QTP uplifts from the mid-Pliocene to the early Pleistocene [65,67,68]. Although our estimations require further testing with additional robust phylogenies and more reliable calibration points, similar diversification times have been reported for weevils (Coleoptera: Curculionidae: *Niphadomimus*) [69] and other plant and animal taxa on the QTP [11,16,26,28,29,70]. The QTP uplift strengthened the East Asia monsoon and increased the aridity of the dry seasons [66], which may have led to the fragmentation of *Gynaephora* populations. Therefore, the *Gynaephora* diversifications may be related to global cooling and desiccation, particularly around the Miocene/Pliocene boundary and during Pleistocene climate fluctuations [71–73]. Thus, our results suggest, together with previous studies, that extensive uplift of the QTP and simultaneous climate changes triggered rapid speciations of many animal taxa on the QTP. However, these correlations require stronger evidence and should be tested in other insects that have high levels of diversity on the QTP.

## Supporting Information

S1 Fig Bayesian phylogenetic trees. (A) the COI dataset, (B) the ND5 dataset, (C) the mitochondrial gene dataset (COI + ND5), (D) the nuclear gene dataset (GAPDH + EF-1α), and (E) the combined dataset (COI + ND5 + GAPDH + EF-1α). Numbers above the branches represent posterior probabilities (PP).
(PDF)

S2 Fig ML phylogenetic trees. (A) the COI dataset, (B) the ND5 dataset, (C) the mitochondrial gene dataset (COI + ND5), (D) the nuclear gene dataset (GAPDH + EF-1α), and (E) the combined dataset (COI + ND5 + GAPDH + EF-1α). Numbers above the branches represent bootstrap support values (BS).
(PDF)

S3 Fig Results of species delimitation using bPTP. (A) the COI dataset, (B) the ND5 dataset, and (C) the mitochondrial gene dataset (COI + ND5). Numbers above the branches represent posterior delimitation probabilities from the Bayesian reconstruction.
(PDF)

S1 Table Detailed information for specimens of *Gynaephora* from the Qinghai-Tibetan Plateau and outgroup taxa included in this study. (XLSX)

S2 Table The best partitioning schemes and models selected by PartitionFinder for each dataset. (DOCX)

S3 Table Detailed information of groups identified by ABGD. (XLSX)

S4 Table Bayes factor analyses of molecular clock and tree models used in BEAST analyses. (DOCX)
