# Supplementary material for: Molecular Phylogeny of Grassland Caterpillars (Lepidoptera: Lymantriinae: Gynaephora) Endemic to the Qinghai-Tibetan Plateau
Source: PLoS One. 2015 Jun 8;10(6):e0127257. doi: 10.1371/journal.pone.0127257 (PMC4459697; doi:10.1371/journal.pone.0127257)

(A) COI_BI

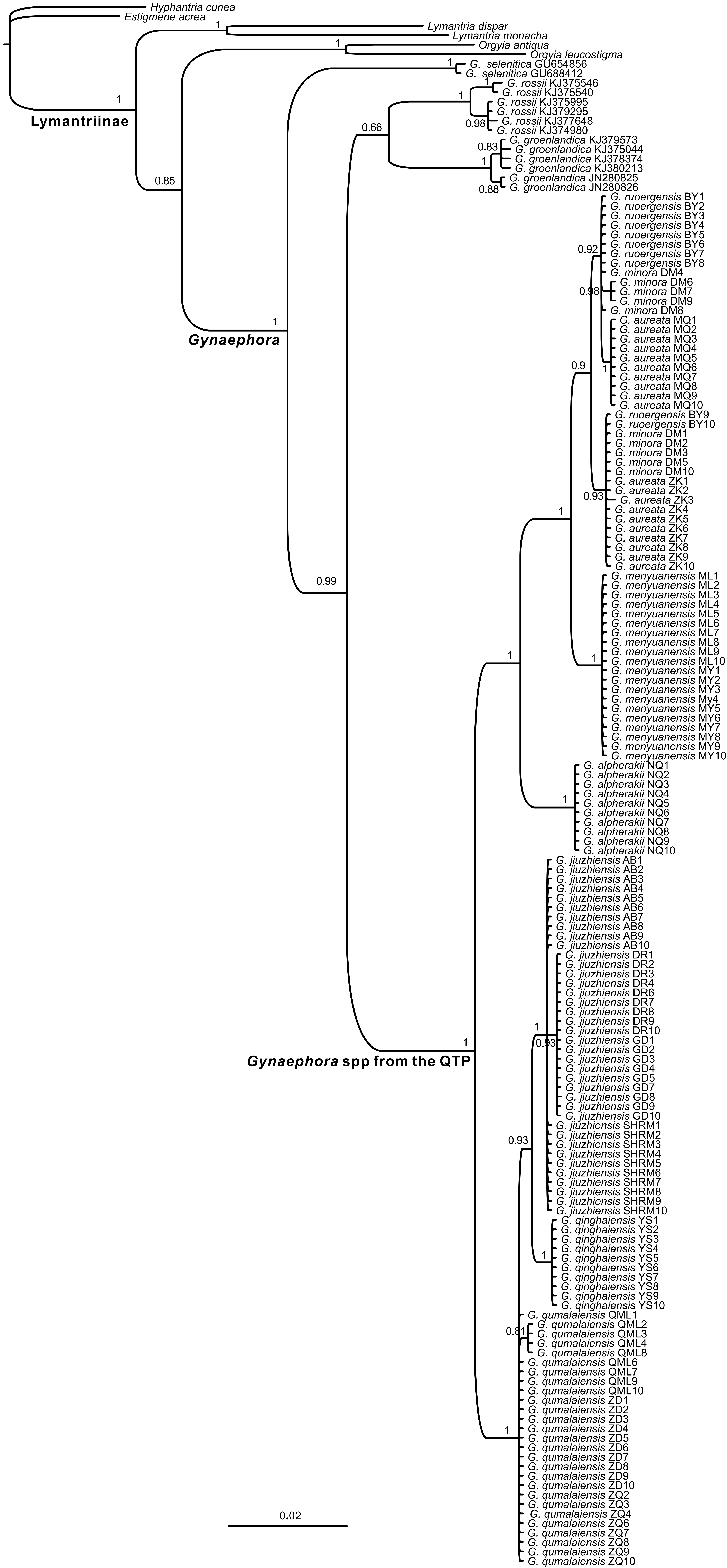

Hyphantria cunea
Estigmene acrea
Lymantria dispar
Lymantria monacha
Orgyia antiqua
Orgyia leucostigma
Lymantriinae
G. selenitica GU654856
G. selenitica GU688412
G. rossii KJ375546
G. rossii KJ375540
G. rossii KJ375995
G. rossii KJ379295
G. rossii KJ377648
G. rossii KJ374980
G. groenlandica KJ379573
G. groenlandica KJ375044
G. groenlandica KJ378374
G. groenlandica KJ380213
G. groenlandica JN280825
G. groenlandica JN280826
Gynaephora
G. ruoergensis BY1
G. ruoergensis BY2
G. ruoergensis BY3
G. ruoergensis BY4
G. ruoergensis BY5
G. ruoergensis BY6
G. ruoergensis BY7
G. ruoergensis BY8
G. minora DM4
G. minora DM6
G. minora DM7
G. minora DM9
G. minora DM8
G. aureata MQ1
G. aureata MQ2
G. aureata MQ3
G. aureata MQ4
G. aureata MQ5
G. aureata MQ6
G. aureata MQ7
G. aureata MQ8
G. aureata MQ9
G. aureata MQ10
G. ruoergensis BY9
G. ruoergensis BY10
G. minora DM1
G. minora DM2
G. minora DM3
G. minora DM5
G. minora DM10
G. aureata ZK1
G. aureata ZK2
G. aureata ZK3
G. aureata ZK4
G. aureata ZK5
G. aureata ZK6
G. aureata ZK7
G. aureata ZK8
G. aureata ZK9
G. aureata ZK10
G. menyuanensis ML1
G. menyuanensis ML2
G. menyuanensis ML3
G. menyuanensis ML4
G. menyuanensis ML5
G. menyuanensis ML6
G. menyuanensis ML7
G. menyuanensis ML8
G. menyuanensis ML9
G. menyuanensis ML10
G. menyuanensis MY1
G. menyuanensis MY2
G. menyuanensis MY3
G. menyuanensis My4
G. menyuanensis MY5
G. menyuanensis MY6
G. menyuanensis MY7
G. menyuanensis MY8
G. menyuanensis MY9
G. menyuanensis MY10
G. alpherakii NQ1
G. alpherakii NQ2
G. alpherakii NQ3
G. alpherakii NQ4
G. alpherakii NQ5
G. alpherakii NQ6
G. alpherakii NQ7
G. alpherakii NQ8
G. alpherakii NQ9
G. alpherakii NQ10
G. jiuzhiensis AB1
G. jiuzhiensis AB2
G. jiuzhiensis AB3
G. jiuzhiensis AB4
G. jiuzhiensis AB5
G. jiuzhiensis AB6
G. jiuzhiensis AB7
G. jiuzhiensis AB8
G. jiuzhiensis AB9
G. jiuzhiensis AB10
G. jiuzhiensis DR1
G. jiuzhiensis DR2
G. jiuzhiensis DR3
G. jiuzhiensis DR4
G. jiuzhiensis DR6
G. jiuzhiensis DR7
G. jiuzhiensis DR8
G. jiuzhiensis DR9
G. jiuzhiensis DR10
G. jiuzhiensis GD1
G. jiuzhiensis GD2
G. jiuzhiensis GD3
G. jiuzhiensis GD4
G. jiuzhiensis GD5
G. jiuzhiensis GD7
G. jiuzhiensis GD8
G. jiuzhiensis GD9
G. jiuzhiensis GD10
G. jiuzhiensis SHRM1
G. jiuzhiensis SHRM2
G. jiuzhiensis SHRM3
G. jiuzhiensis SHRM4
G. jiuzhiensis SHRM5
G. jiuzhiensis SHRM6
G. jiuzhiensis SHRM7
G. jiuzhiensis SHRM8
G. jiuzhiensis SHRM9
G. jiuzhiensis SHRM10
Gynaephora spp from the QTP
G. qinghaiensis YS1
G. qinghaiensis YS2
G. qinghaiensis YS3
G. qinghaiensis YS4
G. qinghaiensis YS5
G. qinghaiensis YS6
G. qinghaiensis YS7
G. qinghaiensis YS8
G. qinghaiensis YS9
G. qinghaiensis YS10
G. qumalaiensis QML1
G. qumalaiensis QML2
G. qumalaiensis QML3
G. qumalaiensis QML4
G. qumalaiensis QML8
G. qumalaiensis QML6
G. qumalaiensis QML7
G. qumalaiensis QML9
G. qumalaiensis QML10
G. qumalaiensis ZD1
G. qumalaiensis ZD2
G. qumalaiensis ZD3
G. qumalaiensis ZD4
G. qumalaiensis ZD5
G. qumalaiensis ZD6
G. qumalaiensis ZD7
G. qumalaiensis ZD8
G. qumalaiensis ZD9
G. qumalaiensis ZD10
G. qumalaiensis ZQ2
G. qumalaiensis ZQ3
G. qumalaiensis ZQ4
G. qumalaiensis ZQ6
G. qumalaiensis ZQ7
G. qumalaiensis ZQ8
G. qumalaiensis ZQ9
G. qumalaiensis ZQ10
0.02

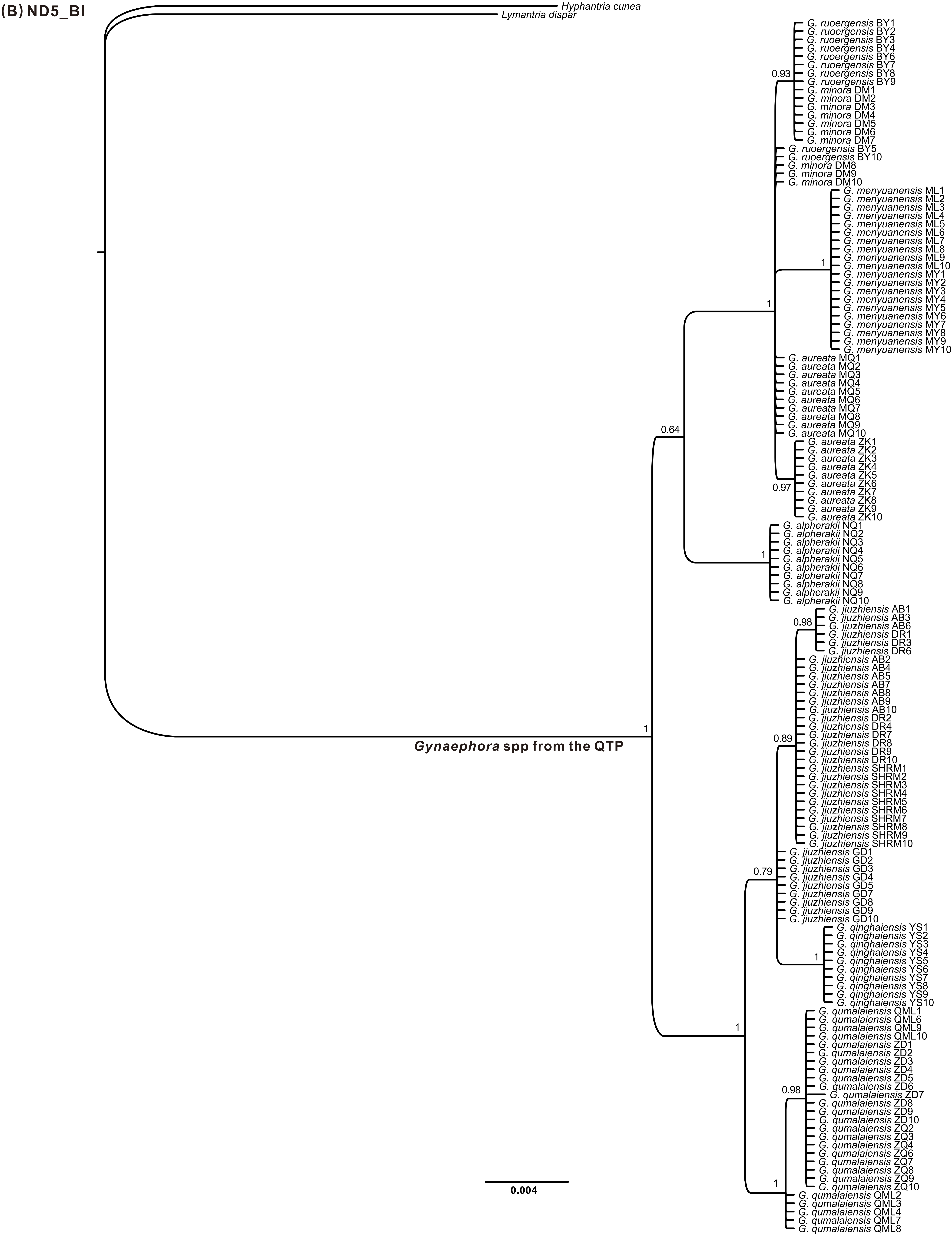
(B) ND5_BI
Hyphantria cunea
Lymantria dispar
G. ruoergensis BY1
G. ruoergensis BY2
G. ruoergensis BY3
G. ruoergensis BY4
G. ruoergensis BY6
G. ruoergensis BY7
G. ruoergensis BY8
G. ruoergensis BY9
G. minora DM1
G. minora DM2
G. minora DM3
G. minora DM4
G. minora DM5
G. minora DM6
G. minora DM7
0.93
G. ruoergensis BY5
G. ruoergensis BY10
G. minora DM8
G. minora DM9
G. minora DM10
G. menyuanensis ML1
G. menyuanensis ML2
G. menyuanensis ML3
G. menyuanensis ML4
G. menyuanensis ML5
G. menyuanensis ML6
G. menyuanensis ML7
G. menyuanensis ML8
G. menyuanensis ML9
G. menyuanensis ML10
G. menyuanensis MY1
G. menyuanensis MY2
G. menyuanensis MY3
G. menyuanensis MY4
G. menyuanensis MY5
G. menyuanensis MY6
G. menyuanensis MY7
G. menyuanensis MY8
G. menyuanensis MY9
G. menyuanensis MY10
1
1
G. aureata MQ1
G. aureata MQ2
G. aureata MQ3
G. aureata MQ4
G. aureata MQ5
G. aureata MQ6
G. aureata MQ7
G. aureata MQ8
G. aureata MQ9
G. aureata MQ10
0.64
G. aureata ZK1
G. aureata ZK2
G. aureata ZK3
G. aureata ZK4
G. aureata ZK5
G. aureata ZK6
G. aureata ZK7
G. aureata ZK8
G. aureata ZK9
G. aureata ZK10
0.97
G. alpherakii NQ1
G. alpherakii NQ2
G. alpherakii NQ3
G. alpherakii NQ4
G. alpherakii NQ5
G. alpherakii NQ6
G. alpherakii NQ7
G. alpherakii NQ8
G. alpherakii NQ9
G. alpherakii NQ10
1
G. jiuzhiensis AB1
G. jiuzhiensis AB3
G. jiuzhiensis AB6
G. jiuzhiensis DR1
G. jiuzhiensis DR3
G. jiuzhiensis DR6
0.98
G. jiuzhiensis AB2
G. jiuzhiensis AB4
G. jiuzhiensis AB5
G. jiuzhiensis AB7
G. jiuzhiensis AB8
G. jiuzhiensis AB9
G. jiuzhiensis AB10
G. jiuzhiensis DR2
G. jiuzhiensis DR4
G. jiuzhiensis DR7
G. jiuzhiensis DR8
G. jiuzhiensis DR9
G. jiuzhiensis DR10
G. jiuzhiensis SHRM1
G. jiuzhiensis SHRM2
G. jiuzhiensis SHRM3
G. jiuzhiensis SHRM4
G. jiuzhiensis SHRM5
G. jiuzhiensis SHRM6
G. jiuzhiensis SHRM7
G. jiuzhiensis SHRM8
G. jiuzhiensis SHRM9
G. jiuzhiensis SHRM10
0.89
1
Gynaephora spp from the QTP
G. jiuzhiensis GD1
G. jiuzhiensis GD2
G. jiuzhiensis GD3
G. jiuzhiensis GD4
G. jiuzhiensis GD5
G. jiuzhiensis GD7
G. jiuzhiensis GD8
G. jiuzhiensis GD9
G. jiuzhiensis GD10
0.79
G. qinghaiensis YS1
G. qinghaiensis YS2
G. qinghaiensis YS3
G. qinghaiensis YS4
G. qinghaiensis YS5
G. qinghaiensis YS6
G. qinghaiensis YS7
G. qinghaiensis YS8
G. qinghaiensis YS9
G. qinghaiensis YS10
1
G. qumalaiensis QML1
G. qumalaiensis QML6
G. qumalaiensis QML9
G. qumalaiensis QML10
G. qumalaiensis ZD1
G. qumalaiensis ZD2
G. qumalaiensis ZD3
G. qumalaiensis ZD4
G. qumalaiensis ZD5
G. qumalaiensis ZD6
1
G. qumalaiensis ZD7
0.98
G. qumalaiensis ZD8
G. qumalaiensis ZD9
G. qumalaiensis ZD10
G. qumalaiensis ZQ2
G. qumalaiensis ZQ3
G. qumalaiensis ZQ4
G. qumalaiensis ZQ6
G. qumalaiensis ZQ7
G. qumalaiensis ZQ8
G. qumalaiensis ZQ9
G. qumalaiensis ZQ10
0.004
1
G. qumalaiensis QML2
G. qumalaiensis QML3
G. qumalaiensis QML4
G. qumalaiensis QML7
G. qumalaiensis QML8

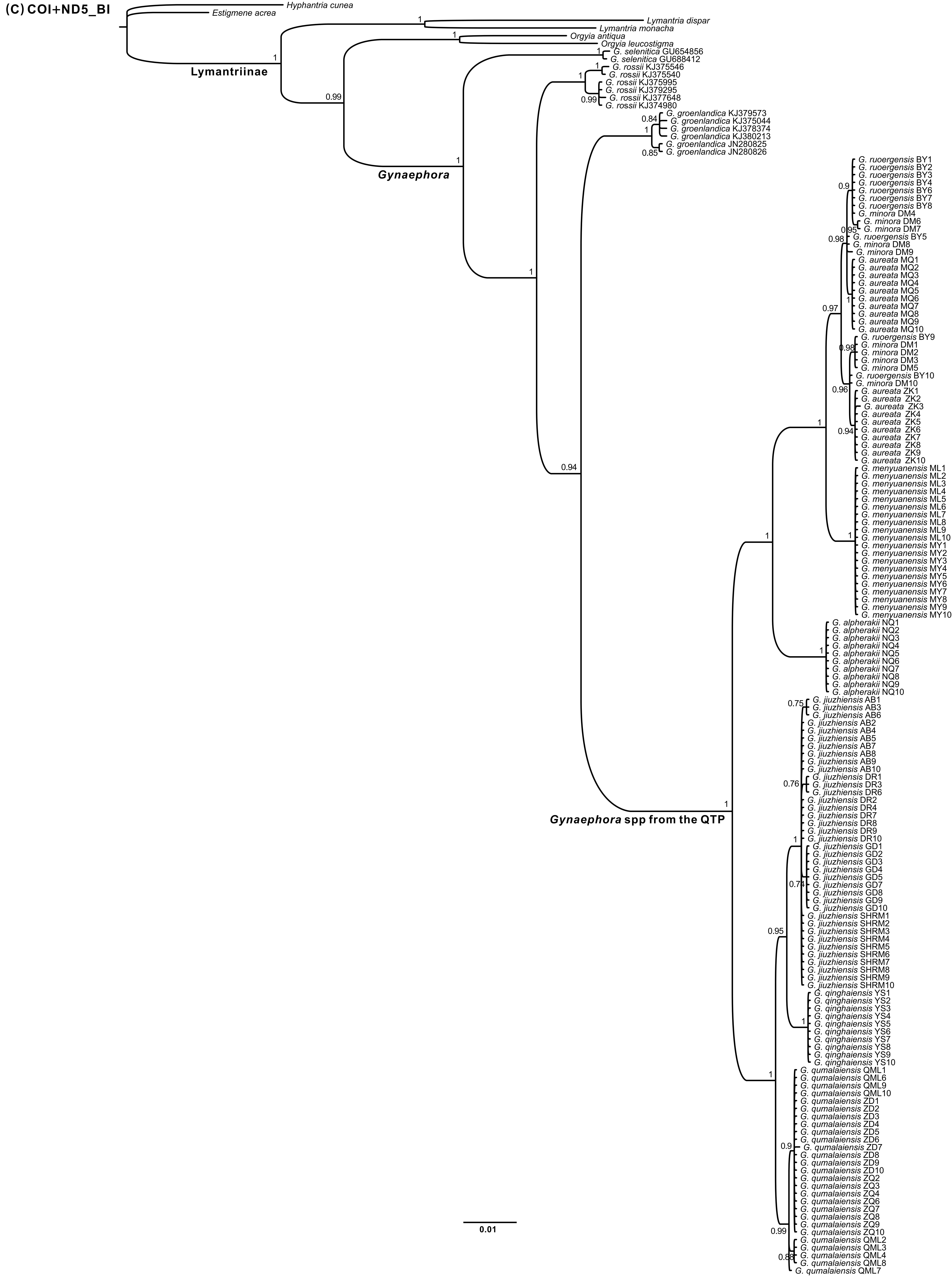
(C) COI+ND5_BI
Hyphantria cunea
Estigmene acrea
Lymantria dispar
Lymantria monacha
Orgyia antiqua
Orgyia leucostigma
Lymantriinae
G. selenitica GU654856
G. selenitica GU688412
G. rossii KJ375546
G. rossii KJ375540
G. rossii KJ375995
G. rossii KJ379295
G. rossii KJ377648
G. rossii KJ374980
G. groenlandica KJ379573
G. groenlandica KJ375044
G. groenlandica KJ378374
G. groenlandica KJ380213
G. groenlandica JN280825
G. groenlandica JN280826
Gynaephora
G. ruoergensis BY1
G. ruoergensis BY2
G. ruoergensis BY3
G. ruoergensis BY4
G. ruoergensis BY6
G. ruoergensis BY7
G. ruoergensis BY8
G. minora DM4
G. minora DM6
G. minora DM7
G. ruoergensis BY5
G. minora DM8
G. minora DM9
G. aureata MQ1
G. aureata MQ2
G. aureata MQ3
G. aureata MQ4
G. aureata MQ5
G. aureata MQ6
G. aureata MQ7
G. aureata MQ8
G. aureata MQ9
G. aureata MQ10
G. ruoergensis BY9
G. minora DM1
G. minora DM2
G. minora DM3
G. minora DM5
G. ruoergensis BY10
G. minora DM10
G. aureata ZK1
G. aureata ZK2
G. aureata ZK3
G. aureata ZK4
G. aureata ZK5
G. aureata ZK6
G. aureata ZK7
G. aureata ZK8
G. aureata ZK9
G. aureata ZK10
G. menyuanensis ML1
G. menyuanensis ML2
G. menyuanensis ML3
G. menyuanensis ML4
G. menyuanensis ML5
G. menyuanensis ML6
G. menyuanensis ML7
G. menyuanensis ML8
G. menyuanensis ML9
G. menyuanensis ML10
G. menyuanensis MY1
G. menyuanensis MY2
G. menyuanensis MY3
G. menyuanensis MY4
G. menyuanensis MY5
G. menyuanensis MY6
G. menyuanensis MY7
G. menyuanensis MY8
G. menyuanensis MY9
G. menyuanensis MY10
G. alpherakii NQ1
G. alpherakii NQ2
G. alpherakii NQ3
G. alpherakii NQ4
G. alpherakii NQ5
G. alpherakii NQ6
G. alpherakii NQ7
G. alpherakii NQ8
G. alpherakii NQ9
G. alpherakii NQ10
G. jiuzhiensis AB1
G. jiuzhiensis AB3
G. jiuzhiensis AB6
G. jiuzhiensis AB2
G. jiuzhiensis AB4
G. jiuzhiensis AB5
G. jiuzhiensis AB7
G. jiuzhiensis AB8
G. jiuzhiensis AB9
G. jiuzhiensis AB10
G. jiuzhiensis DR1
G. jiuzhiensis DR3
G. jiuzhiensis DR6
G. jiuzhiensis DR2
G. jiuzhiensis DR4
G. jiuzhiensis DR7
G. jiuzhiensis DR8
G. jiuzhiensis DR9
G. jiuzhiensis DR10
G. jiuzhiensis GD1
G. jiuzhiensis GD2
G. jiuzhiensis GD3
G. jiuzhiensis GD4
G. jiuzhiensis GD5
G. jiuzhiensis GD7
G. jiuzhiensis GD8
G. jiuzhiensis GD9
G. jiuzhiensis GD10
G. jiuzhiensis SHRM1
G. jiuzhiensis SHRM2
G. jiuzhiensis SHRM3
G. jiuzhiensis SHRM4
G. jiuzhiensis SHRM5
G. jiuzhiensis SHRM6
G. jiuzhiensis SHRM7
G. jiuzhiensis SHRM8
G. jiuzhiensis SHRM9
G. jiuzhiensis SHRM10
Gynaephora spp from the QTP
G. qinghaiensis YS1
G. qinghaiensis YS2
G. qinghaiensis YS3
G. qinghaiensis YS4
G. qinghaiensis YS5
G. qinghaiensis YS6
G. qinghaiensis YS7
G. qinghaiensis YS8
G. qinghaiensis YS9
G. qinghaiensis YS10
G. qumalaiensis QML1
G. qumalaiensis QML6
G. qumalaiensis QML9
G. qumalaiensis QML10
G. qumalaiensis ZD1
G. qumalaiensis ZD2
G. qumalaiensis ZD3
G. qumalaiensis ZD4
G. qumalaiensis ZD5
G. qumalaiensis ZD6
G. qumalaiensis ZD7
G. qumalaiensis ZD8
G. qumalaiensis ZD9
G. qumalaiensis ZD10
G. qumalaiensis ZQ2
G. qumalaiensis ZQ3
G. qumalaiensis ZQ4
G. qumalaiensis ZQ6
G. qumalaiensis ZQ7
G. qumalaiensis ZQ8
G. qumalaiensis ZQ9
G. qumalaiensis ZQ10
G. qumalaiensis QML2
G. qumalaiensis QML3
G. qumalaiensis QML4
G. qumalaiensis QML8
G. qumalaiensis QML7
0.01

(D) EF+GAPDH_BI

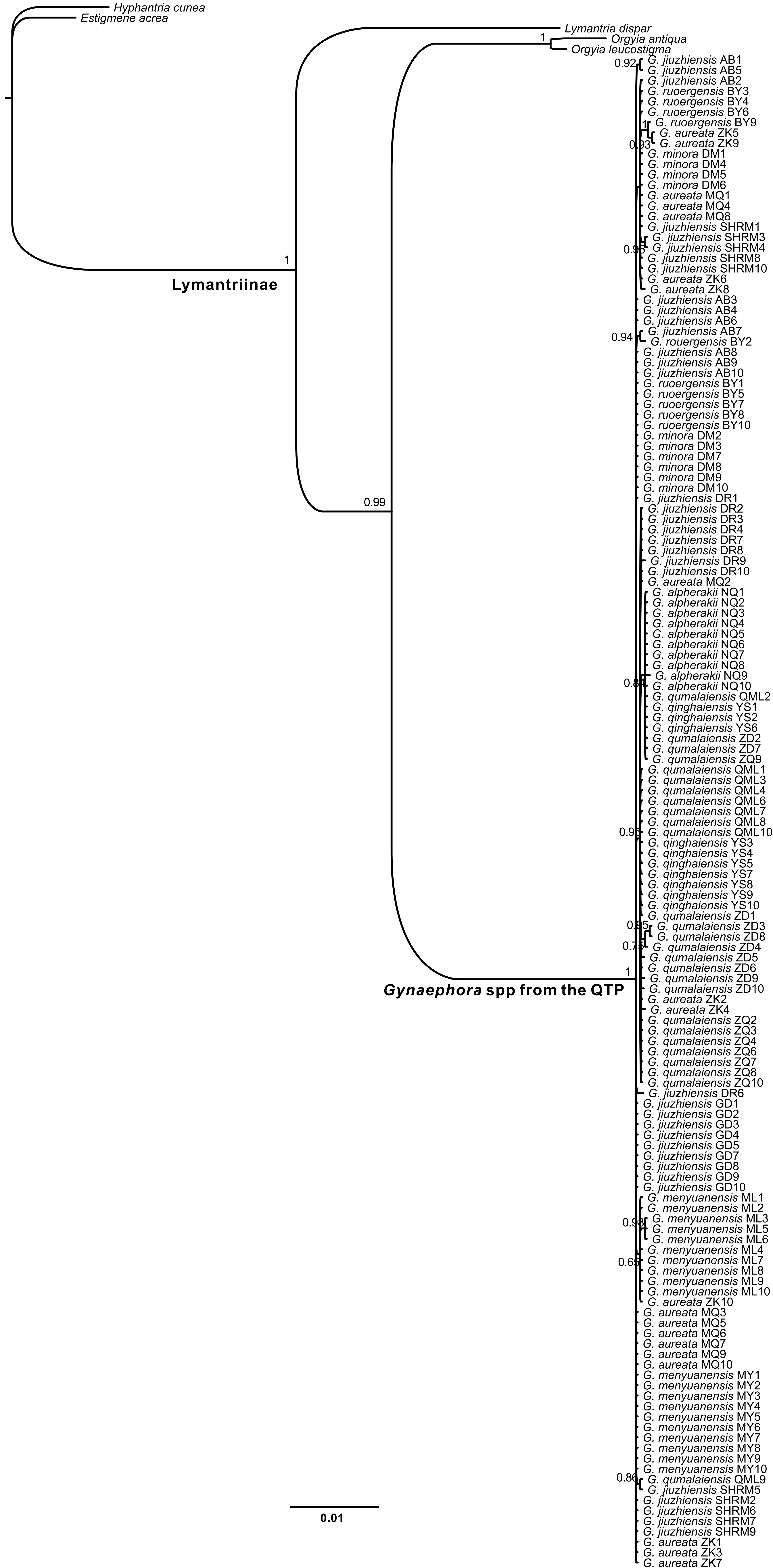

Hyphantria cunea
Estigmene acrea
Lymantria dispar
Orgyia antiqua
Orgyia leucostigma
1
Lymantriinae
0.99
Gynaephora spp from the QTP
G. jiuzhiensis AB1
G. jiuzhiensis AB5
G. jiuzhiensis AB2
G. ruoergensis BY3
G. ruoergensis BY4
G. ruoergensis BY6
G. ruoergensis BY9
G. aureata ZK5
G. aureata ZK9
G. minora DM1
G. minora DM4
G. minora DM5
G. minora DM6
G. aureata MQ1
G. aureata MQ4
G. aureata MQ8
G. jiuzhiensis SHRM1
G. jiuzhiensis SHRM3
G. jiuzhiensis SHRM4
G. jiuzhiensis SHRM8
G. jiuzhiensis SHRM10
G. aureata ZK6
G. aureata ZK8
G. jiuzhiensis AB3
G. jiuzhiensis AB4
G. jiuzhiensis AB6
G. jiuzhiensis AB7
G. rouergensis BY2
G. jiuzhiensis AB8
G. jiuzhiensis AB9
G. jiuzhiensis AB10
G. ruoergensis BY1
G. ruoergensis BY5
G. ruoergensis BY7
G. ruoergensis BY8
G. ruoergensis BY10
G. minora DM2
G. minora DM3
G. minora DM7
G. minora DM8
G. minora DM9
G. minora DM10
G. jiuzhiensis DR1
G. jiuzhiensis DR2
G. jiuzhiensis DR3
G. jiuzhiensis DR4
G. jiuzhiensis DR7
G. jiuzhiensis DR8
G. jiuzhiensis DR9
G. jiuzhiensis DR10
G. aureata MQ2
G. alpherakii NQ1
G. alpherakii NQ2
G. alpherakii NQ3
G. alpherakii NQ4
G. alpherakii NQ5
G. alpherakii NQ6
G. alpherakii NQ7
G. alpherakii NQ8
G. alpherakii NQ9
G. alpherakii NQ10
G. qumalaiensis QML2
G. qinghaiensis YS1
G. qinghaiensis YS2
G. qinghaiensis YS6
G. qumalaiensis ZD2
G. qumalaiensis ZD7
G. qumalaiensis ZQ9
G. qumalaiensis QML1
G. qumalaiensis QML3
G. qumalaiensis QML4
G. qumalaiensis QML6
G. qumalaiensis QML7
G. qumalaiensis QML8
G. qumalaiensis QML10
G. qinghaiensis YS3
G. qinghaiensis YS4
G. qinghaiensis YS5
G. qinghaiensis YS7
G. qinghaiensis YS8
G. qinghaiensis YS9
G. qinghaiensis YS10
G. qumalaiensis ZD1
G. qumalaiensis ZD3
G. qumalaiensis ZD8
G. qumalaiensis ZD4
G. qumalaiensis ZD5
G. qumalaiensis ZD6
G. qumalaiensis ZD9
G. qumalaiensis ZD10
G. aureata ZK2
G. aureata ZK4
G. qumalaiensis ZQ2
G. qumalaiensis ZQ3
G. qumalaiensis ZQ4
G. qumalaiensis ZQ6
G. qumalaiensis ZQ7
G. qumalaiensis ZQ8
G. qumalaiensis ZQ10
G. jiuzhiensis DR6
G. jiuzhiensis GD1
G. jiuzhiensis GD2
G. jiuzhiensis GD3
G. jiuzhiensis GD4
G. jiuzhiensis GD5
G. jiuzhiensis GD7
G. jiuzhiensis GD8
G. jiuzhiensis GD9
G. jiuzhiensis GD10
G. menyuanensis ML1
G. menyuanensis ML2
G. menyuanensis ML3
G. menyuanensis ML5
G. menyuanensis ML6
G. menyuanensis ML4
G. menyuanensis ML7
G. menyuanensis ML8
G. menyuanensis ML9
G. menyuanensis ML10
G. aureata ZK10
G. aureata MQ3
G. aureata MQ5
G. aureata MQ6
G. aureata MQ7
G. aureata MQ9
G. aureata MQ10
G. menyuanensis MY1
G. menyuanensis MY2
G. menyuanensis MY3
G. menyuanensis MY4
G. menyuanensis MY5
G. menyuanensis MY6
G. menyuanensis MY7
G. menyuanensis MY8
G. menyuanensis MY9
G. menyuanensis MY10
G. qumalaiensis QML9
G. jiuzhiensis SHRM5
G. jiuzhiensis SHRM2
G. jiuzhiensis SHRM6
G. jiuzhiensis SHRM7
G. jiuzhiensis SHRM9
G. aureata ZK1
G. aureata ZK3
G. aureata ZK7
0.01

(E) Four genes_BI

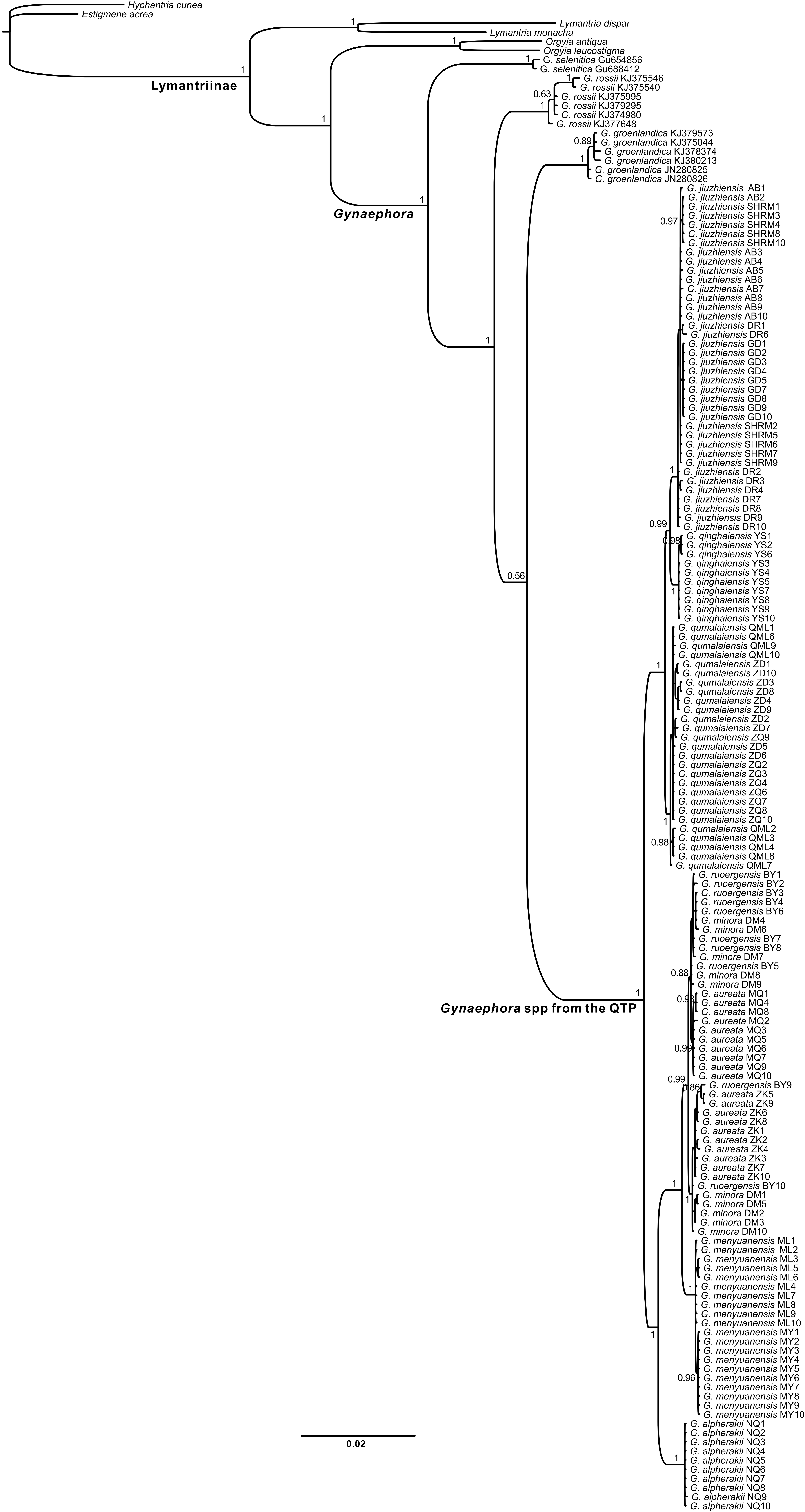

Supplement: S1 Fig — (A) the COI dataset, (B) the ND5 dataset, (C) the mitochondrial gene dataset (COI + ND5), (D) the nuclear gene dataset (GAPDH + EF-1α), and (E) the combined dataset (COI + ND5 + GAPDH + EF-1α). Numbers above the branches represent posterior probabilities (PP). (PDF) [file pone.0127257.s001.pdf]
