# Supplementary figures and images for: Molecular Phylogeny of Grassland Caterpillars (Lepidoptera: Lymantriinae: Gynaephora) Endemic to the Qinghai-Tibetan Plateau
Source: PLoS One. 2015 Jun 8;10(6):e0127257. doi: 10.1371/journal.pone.0127257 (PMC4459697; doi:10.1371/journal.pone.0127257)

(A) COI\_ML

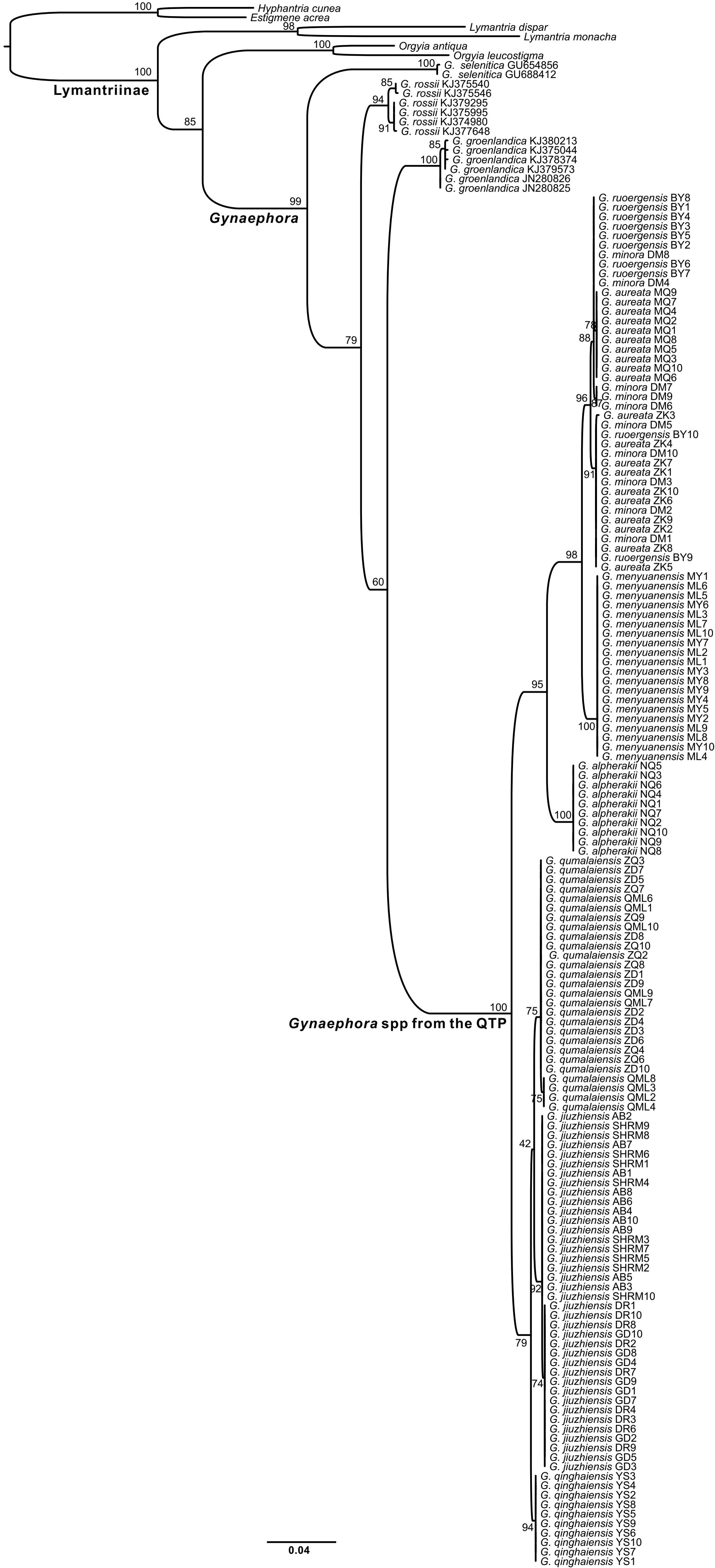

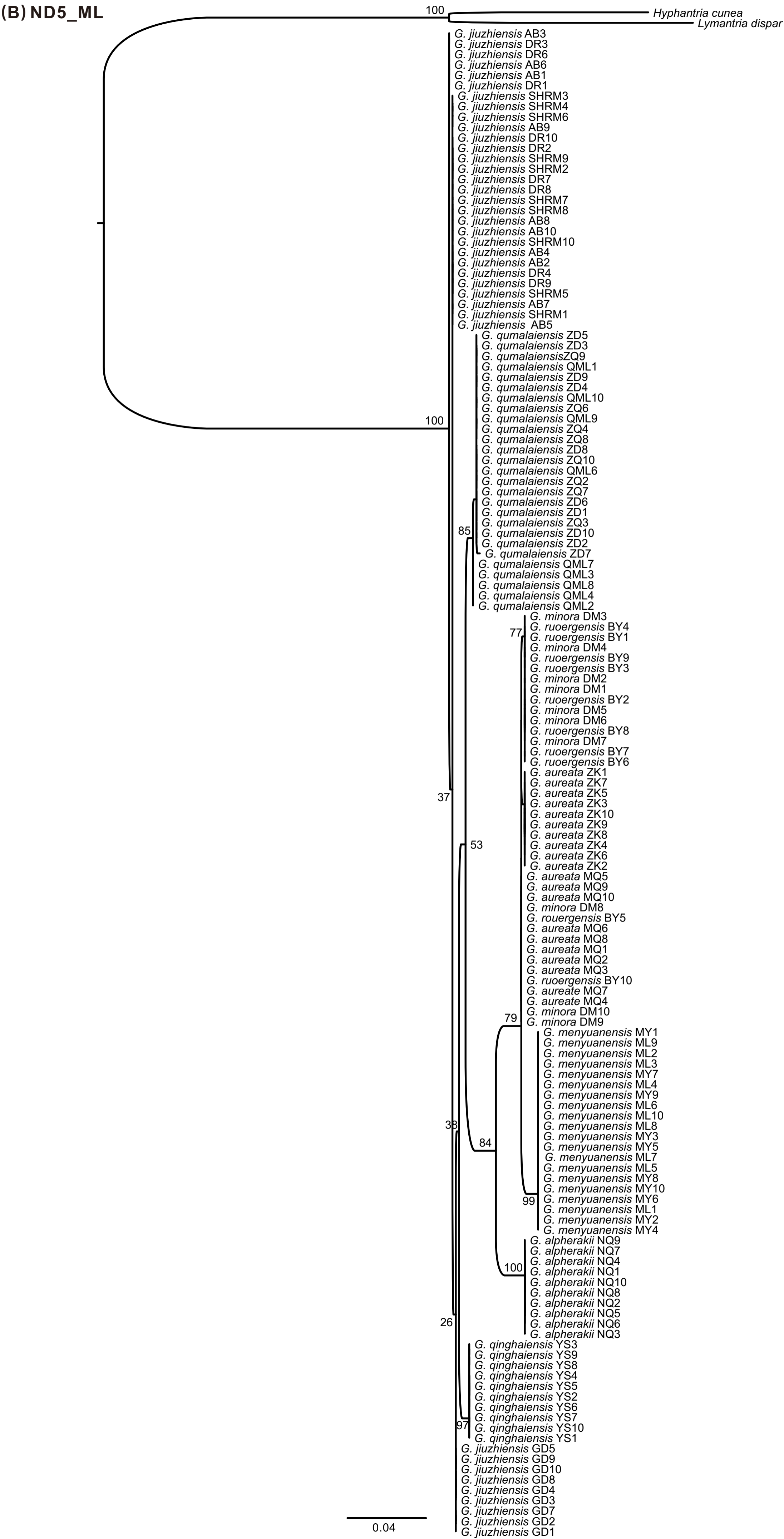

(C) COI+ND5\_ML

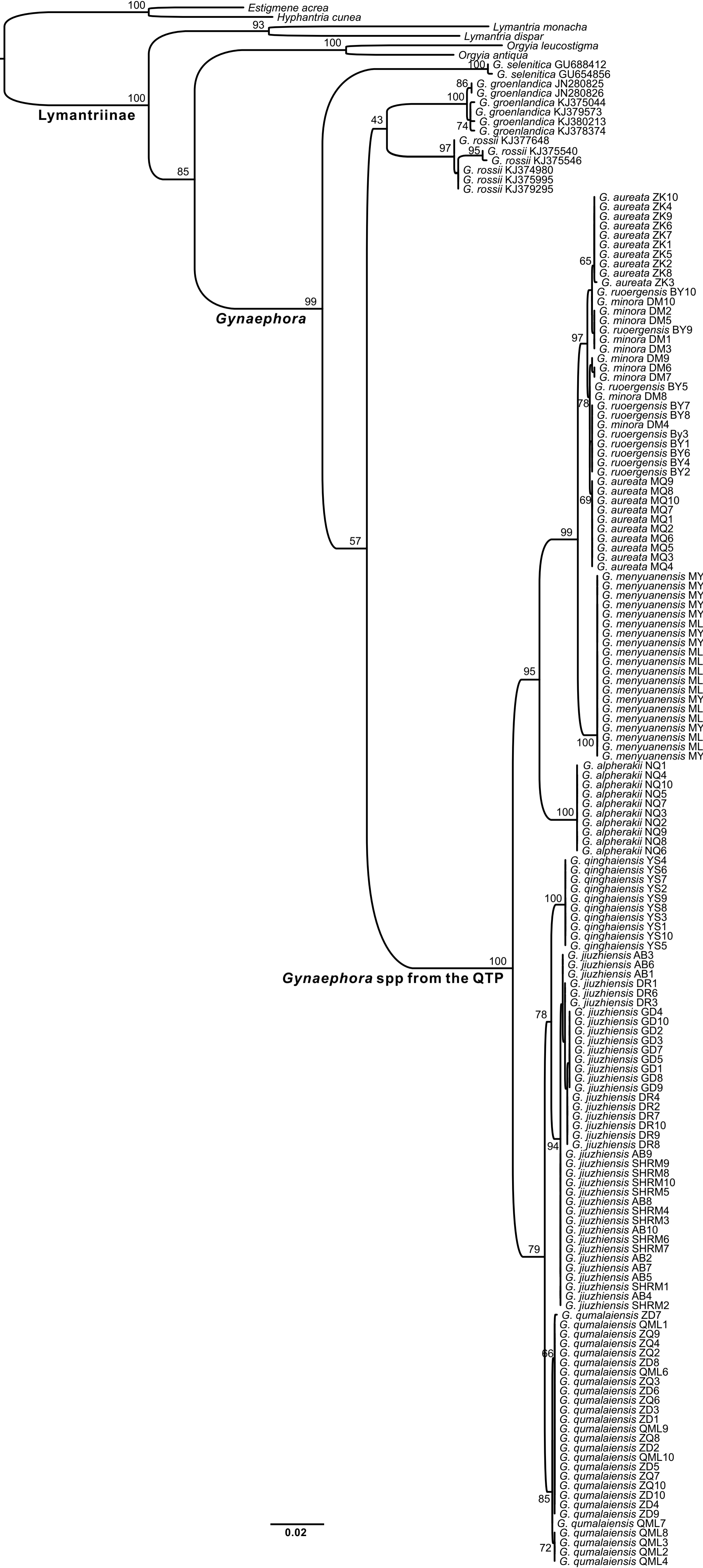

(D) EF+GAPDH\_ML

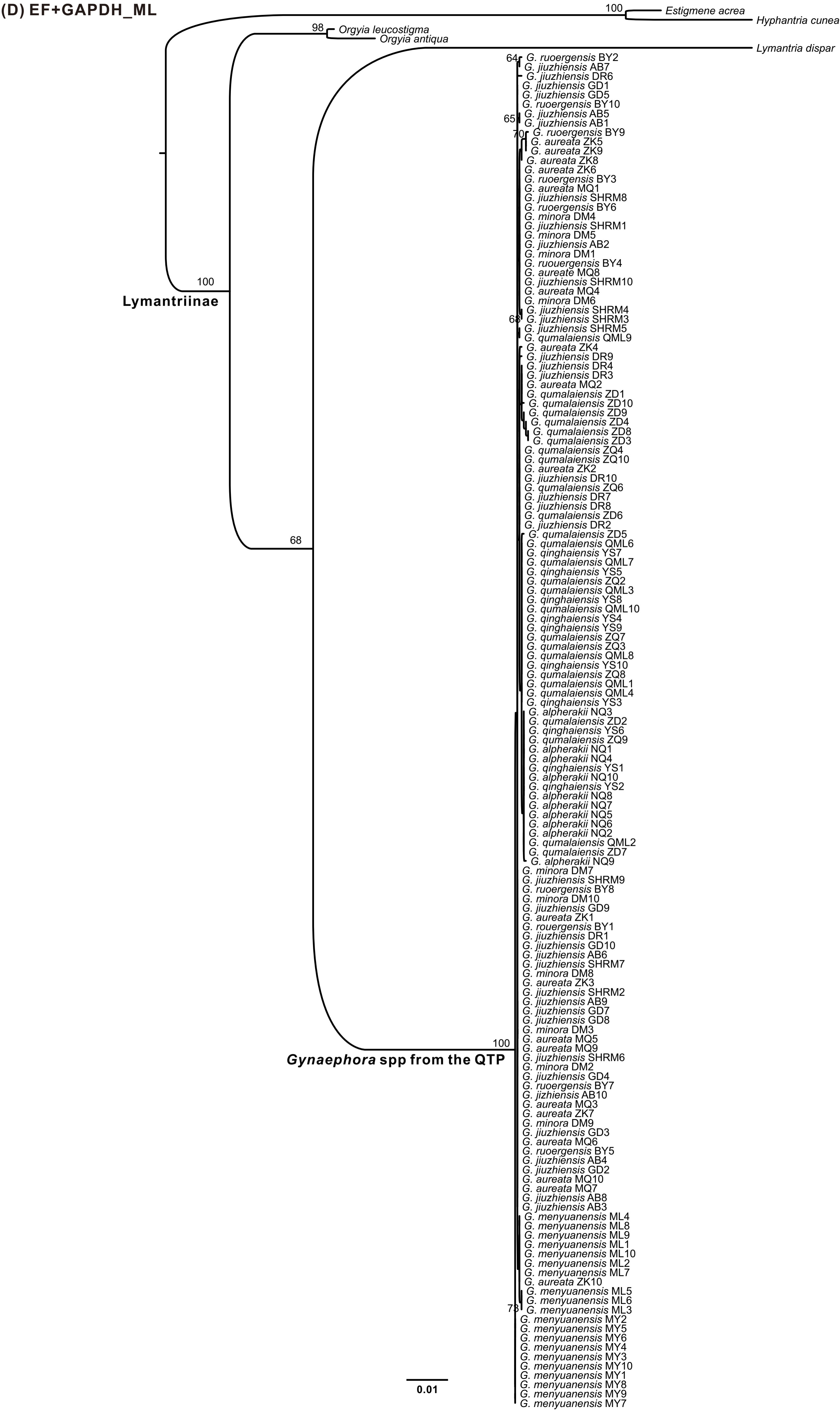

(E) Four genes\_ML

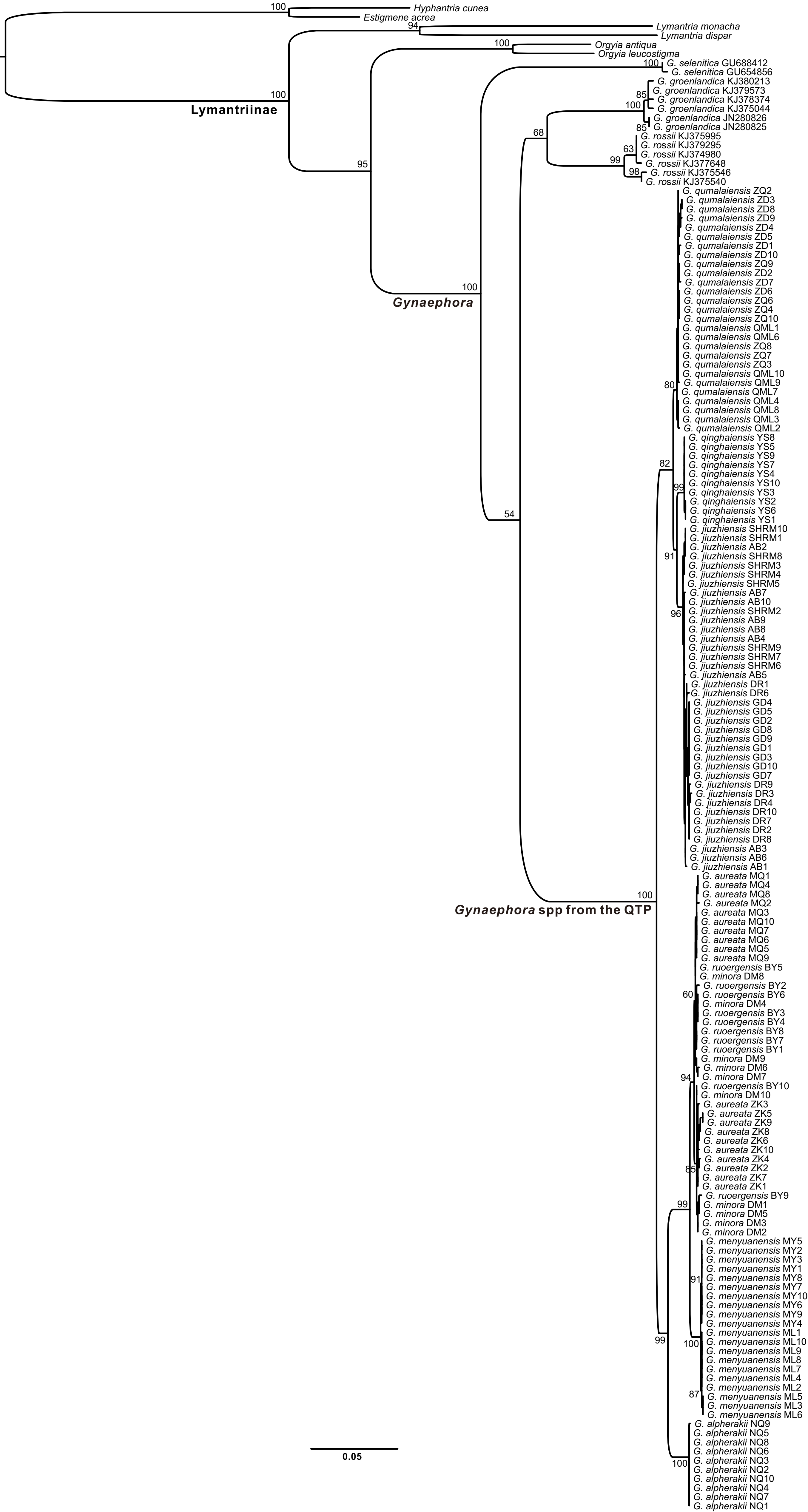

Supplement: S2 Fig — (A) the COI dataset, (B) the ND5 dataset, (C) the mitochondrial gene dataset (COI + ND5), (D) the nuclear gene dataset (GAPDH + EF-1α), and (E) the combined dataset (COI + ND5 + GAPDH + EF-1α). Numbers above the branches represent bootstrap support values (BS). (PDF) [file pone.0127257.s002.pdf]

(A) COI

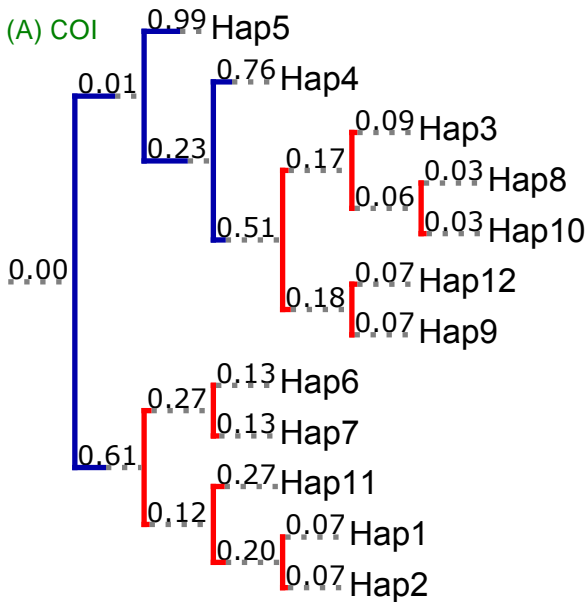

0.10

(B) ND5

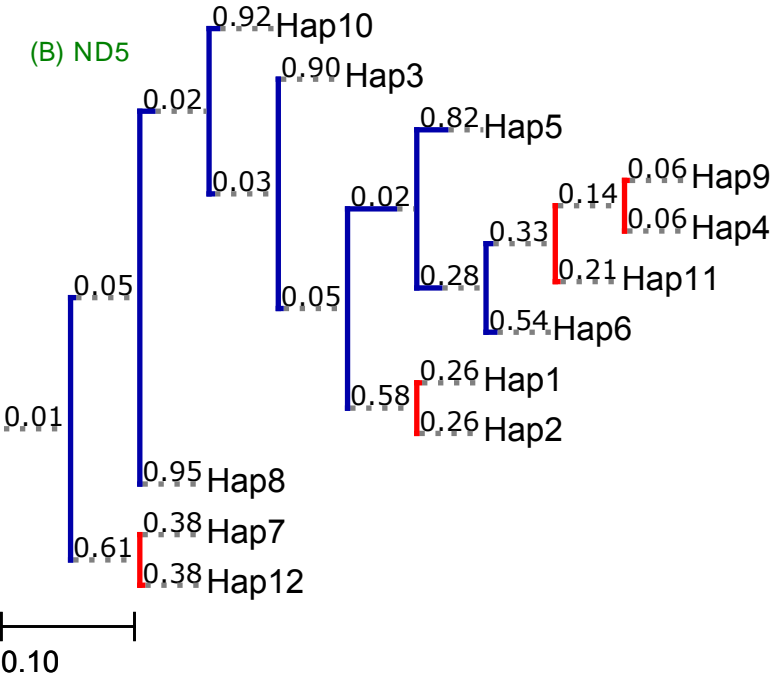

(C) COI+ND5

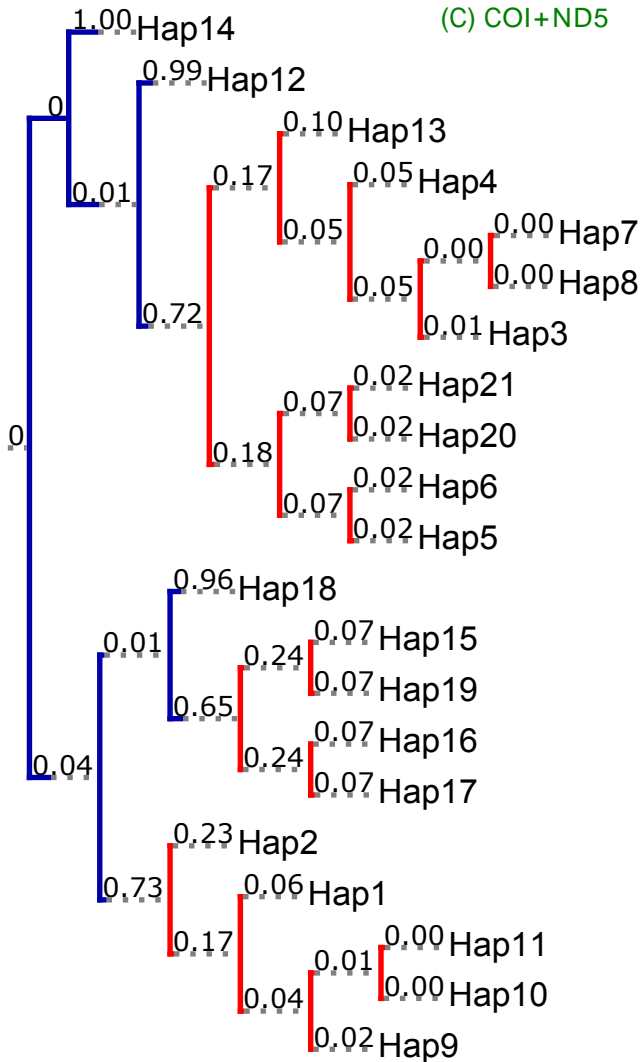

Supplement: S3 Fig — (A) the COI dataset, (B) the ND5 dataset, and (C) the mitochondrial gene dataset (COI + ND5). Numbers above the branches represent posterior delimitation probabilities from the Bayesian reconstruction. (PDF) [file pone.0127257.s003.pdf]
