# Supplementary material for: Molecular Phylogeny of Grassland Caterpillars (Lepidoptera: Lymantriinae: Gynaephora) Endemic to the Qinghai-Tibetan Plateau
Source: PLoS One. 2015 Jun 8;10(6):e0127257. doi: 10.1371/journal.pone.0127257 (PMC4459697; doi:10.1371/journal.pone.0127257)
Supplement: S2 Table — (DOCX) [file pone.0127257.s005.docx]

**Table S2.** The best partitioning schemes and models selected by PartitionFinder for each dataset.

| Dataset | Number of subsets | Subset Partitions | Best Model |
| --- | --- | --- | --- |
| COI | 1 | COI_pos1, COI_pos2, COI_pos3 | GTR+G |
| ND5 | 1 | ND5_pos1, ND5_pos2, ND5_pos3 | GTR+I |
| EF | 1 | EF_pos1, EF_pos2, EF_pos3 | SYM+G |
| GAPDH | 1 | GAPDH_pos1, GAPDH_pos2, GAPDH_pos3 | K80 |
| COI+ND5 | 1 | COI_pos1, COI_pos2, COI_pos3, ND5_pos1, ND5_pos2, ND5_pos3 | GTR+G |
| EF+GAPDH | 1 | EF_pos1, EF_pos2, EF_pos3, GAPDH_pos1, GAPDH_pos2, GAPDH_pos3 | SYM+G |
| COI+ND5+EF+GAPDH | 1 | COI_pos1, COI_pos2, COI_pos3, EF_pos1, EF_pos2, EF_pos3, GAPDH_pos1, GAPDH_pos2, GAPDH_pos3, ND5_pos1, ND5_pos2, ND5_pos3 | GTR+I+G |
