# Supplementary material for: Molecular Phylogeny of Grassland Caterpillars (Lepidoptera: Lymantriinae: Gynaephora) Endemic to the Qinghai-Tibetan Plateau
Source: PLoS One. 2015 Jun 8;10(6):e0127257. doi: 10.1371/journal.pone.0127257 (PMC4459697; doi:10.1371/journal.pone.0127257)
Supplement: S4 Table — (DOCX) [file pone.0127257.s007.docx]

**Table S4.** Bayes factor analyses of molecular clock and tree models used in BEAST analysis.

| Model | | Marginal likelihood | |
| --- | --- | --- | --- |
|  |  | PS | SS |
| Lognormal relaxed clock | Birth-Death process | -15142.8* | -15142.6* |
|  | Yule process | -15743.7 | -15743.7 |
| Birth-Death process | Strict clock | -16287.3 | -16287.3 |
|  | Lognormal relaxed clock | -15142.8* | -15142.6* |
|  | Exponential relaxed clock | -16240.2 | -16240.1 |

The log marginal likelihood value for each model is listed in the table, and the preferred model is labeled with asterisk. PS = path sampling; SS = stepping-stone sampling.
